# Sustainable approach of La doped CuFe_2_O_4_ nanomaterial for electrochemical lead and paracetamol sensing action with multiple applications

**DOI:** 10.1038/s41598-023-45029-y

**Published:** 2023-10-19

**Authors:** Meenakshi Giridhar, B. C. Manjunath, B. S. Surendra, K. N. Harish, S. C. Prashantha, T. Kiran, B. Uma, H. C. Ananda Murthy

**Affiliations:** 1 Department of Physics, St. Phelomena’s College, University of Mysore, Mysore, India; 2 Department of Physics, Yuvaraja’s College, University of Mysore, Mysore, India; 3 Department of Chemistry, Dayananda Sagar College of Engineering, Bangalore, 560111 India; 4 Department of Chemistry, BMS College of Engineering, Bull Temple Road, Bangalore, 560019 India; 5 Taproot PU College Yelahanka, Bangalore, India; 6 Department of Chemistry, SJB Institute of Technology, Bangalore, 560 060 India; 7 Department of Applied Chemistry, School of Applied Natural Science, Adama Science and Technology University, P O Box 1888, Adama, Ethiopia; 8 Department of Prosthodontics, Saveetha Dental College & Hospital, Saveetha Institute of Medical and Technical Sciences (SIMATS), Saveetha University, Chennai, 600077 Tamil Nadu India

**Keywords:** Chemistry, Nanoscience and technology

## Abstract

This present research aimed to investigate the novel applications of synthesized La doped CuFe_2_O_4_ nanomaterial (LCF NMs) using renewable bio-fuel (*Aegle Marmelos extract*) by combustion process. The sensor applications were accomplished by modified electrode using LCF NMs with graphite powder and examined its excellent sensing action towards heavy metal (Lead content) and drug chemical (Paracetamol) substances. The thermodynamics of redox potential and super-capacitor behavior of LCF NMs were investigated through Cyclic Voltametric (CV) and Electrochemical Impedance Spectral (EIS) methods under specific conditions at scan rate of 1 to 5 mV/s. The heterogeneous photo-catalytic process of prepared NMs on Fast orange Red (FOR) dye-decolouration was investigated and noted its excellent degradation (91.7%) at 90 min using 20 ppm of dye solution and 40 mg of synthesized samples under Sun-light irradiation. Further, the antibacterial activity of synthesized NMs is investigated against various strains of gram positive (*Bacillus subtillis*) and gram negative bacteria (*Pseudomonas aeruginosa*), which confirms that the LCF NMs have higher activity towards gram positive bacteria with an average inhibition zone of 19 mm. This synthesized LCF NMs is a multi-functional material with stable and eco-friendly materials.

## Introduction

Nanomaterials have received a potential attraction to researchers owing to its cost-effective, persistence, optoelectronics, sustainable-energy source, catalyst activity, and other environmental concerns^1–4^. However, the rapid increase of human population and development of global economy has leads to the increasing lack of energy associated resources^5,6^. Thus, the eco-friendly supercapacitor energy source are drawing great attention towards fast charging-discharging capability, higher cycle-life and power density^7,8^. Currently, the several nanomaterials viz; MnFe_2_O_4_, NiCo_2_O_4_, CuFe_2_O_4,_ CoFe_2_O_4_, NiFe_2_O_4_, ZnFe_2_O_4_, MnCo_2_O_4_, etc., have been widely examined for their supercapacitor characterizations^9–14^. Among the different kind of nanomaterials, lanthanum doped metal ferrite (La-CuFe_2_O_4_) NPs with spinel structure and its general formula was represented as X_x_Y_3−x_O_4_, here X and Y are two specific transition/inner transition metal types have demonstrated electrochemical storage characterizations. The La–CuFe_2_O_4_ NPs is an interesting spinel ferrite due to its physicochemical properties and potential multi-applications viz; chemical/bio-sensors, energy storage and intensive photocatalyst for clean energy in ecological action. These La ion based ferrite nanoparticles shows well performance towards electrochemical reactions would support the battery-energy storage activity with longer charging-discharging cycles.

In recent years, the nanomaterials are most prominent object and drawn its attentions for researchers towards synthesis, design and multiple-applications due to its unique physico-chemical characteristics such as optical, catalytic, thermal, electrical, magnetic, etc.^15–17^. the survey of the nanomaterials shows an excellent photocatalytic activities under visible and Sun light irradiation. Hence, the nanomaterials were successfully utilized for degradation of toxic effluents discharged from the fast population growth and industrialization activities to prevention of water pollution^18^. Among the wide range of nanomaterials, nano-ferrite has gained great attention towards prevention of such kind of environmentally issues. Biomass materials are renewable resources and attracting great attention towards various applications such as catalytic activity, fuel for nanomaterial and organic synthesis, etc. The Aegle marmelos biomass material has a wide range of microbial applications like anti-bacterial, anti-diarrheal, anti-diabetic, antiviral etc. Nowadays, the various kinds of fuels are utilized for nanomaterial synthesis under solution-combustion process^19^. During the nanoparticle synthesis, fuels are actively involved in combustion process and discharged huge quantity of heat and harmful gases. Therefore, the green bio-materials (plant extracts) were used as a alternate to chemical assisted preparation due to its action as reducing and capping agents in preparation of nanoparticles.

The literature survey of ferrite based nanomaterial reveals that several electrochemical reactions have been reported, such as Giridhar meenakshi et al.^20^ has described a very good performance towards electrochemical investigation by cyclic voltammetry analysis of CuFe_2_O_4_ nanoparticle. Lakshmi ranganatha et al.^21^ has reported on preparation of ZnFe_2_O_4_ NPs with supercapacitor nature and high specific capacitance. Raghavendra et al.^22^ has described as an excellent electrochemical performance observed by its reduction–oxidation potential. Ahmed et al.^23^ has reported the effect of La^3+^ doping on MgFe_2_O_4_ NPs, which shows ortho-ferrite phase formation and enhancing activity in electrical with magnetic properties. Almessiere et al.^24^ revealed that La^3+^ doped CuFe_2_O_4_ NPs has A.C. conductivity decreased due to the increasing La^3+^ ions concentration. The doping of metal ions into lattices of copper ferrite with a specific metal cations leads to changes the energy band gap with electrical characteristics of doped nanomaterial^25^. Hence, our present reported research work mainly focused on additional implementation in the electrochemical investigations of La–CuFe_2_O_4_ NPs. This research work done systematically their examinations on LCF NMs synthesized from green-fuel mediated solution combustion process and well characterized from various spectral techniques viz XRD, SEM–EDX, TEM, FT-IR, BET and DRS spectral techniques. The obtained electrochemical results confirms that higher redox reaction performance and supercapacitor behaviour examined by CV and EIS studies. Further, the modified electrode by LCF with graphite is implemented to most significant electrochemical sensor detection of paracetamol drug molecule and heavy metal (Lead content) in 0.1 M HCl electrolytic solution. Additionally, the excellent photocatalytic dye degradation and microbial examinations were recorded for achieved LCF NMs.

## Experimental

### Activity of AME green fuel preparation

The non-edible *Aegle Marmelos* fruits were collected from Boppanahalli village, H D Kote, Mysore, Karnataka, India. The inner part of AME fruits were separated and allowed to grinding with help of grinder mixer, resulted crude liquid was subjected to filtration using Whatman filter paper and utilized as green-fuel for synthesis of LCF NMs.

### Synthetic procedure of LCF NMs

**Figure 1:**
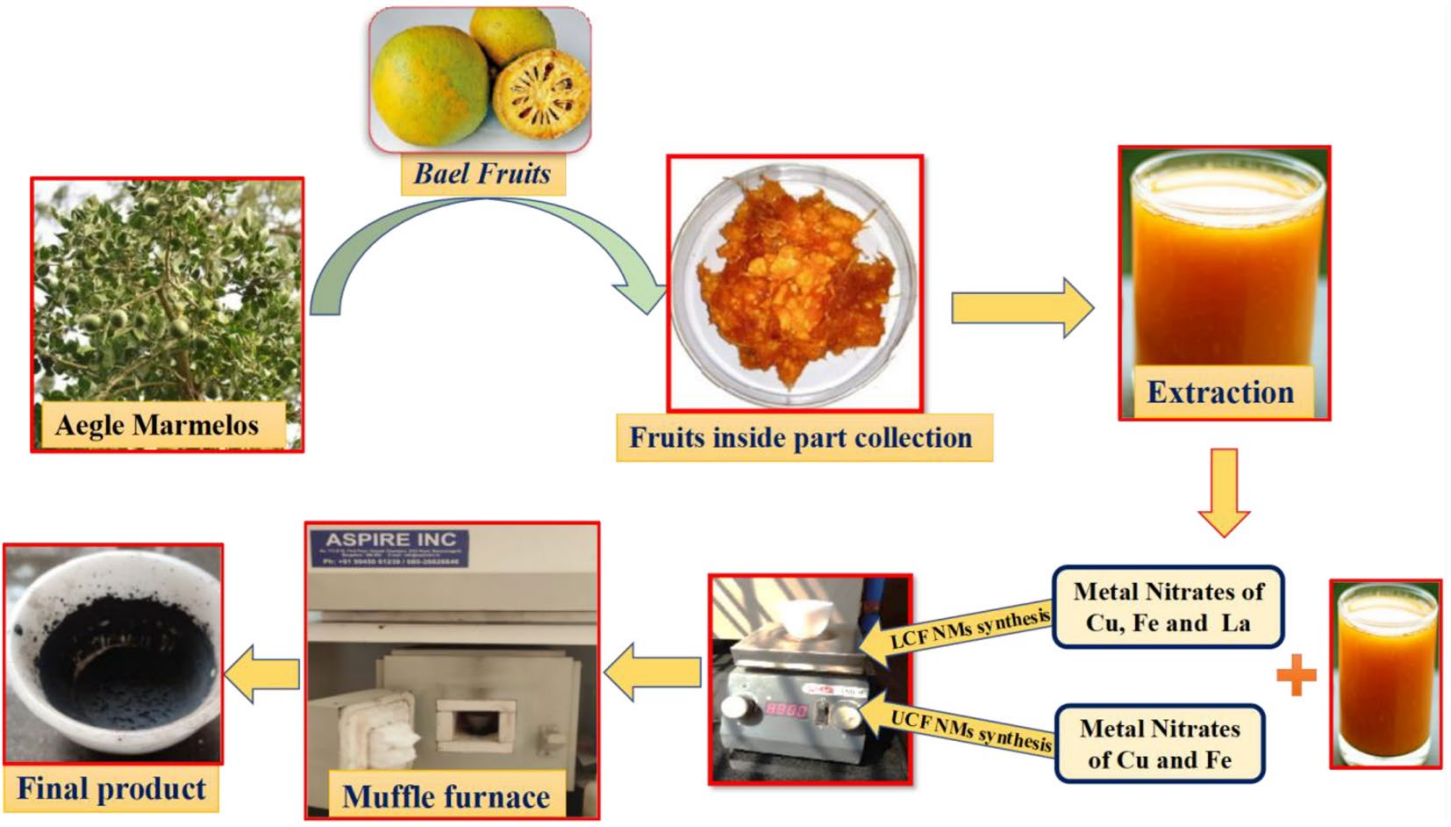
The experimental preparation steps of synthesis of UCF and LCF NMs.

The synthetic procedure of LCF NMs is enclosed with stochiometric ratios of initial chemical reagents are 0.0544 g of Cu(NO_3_)_2_·3H_2_O (0.0003 mol), 3.298 g of Fe(NO_3_)_2_·9H_2_O (0.014 mol), 0.472 g of La(NO_3_)_3_ (0.0015 mol), (Fine Chemicals Company Ltd.) and laboratory prepared *Aegle Marmelos extract* as a green fuel used without further purification. The specific amount of above metal nitrates and optimized 3 mL green fuel were taken in silica crucible and subjected to mechanical stirring for attaining homogeneity. Further, this solution mixture was subjected to combustion process by placing into the muffle furnace maintained temperature at 450 ± 10 °C. Finally, the obtained blackish nanoparticle was collected and used for material confirmation by various spectral characterizations. Similarly, the host CuFe_2_O_4_ NMs was prepared by following the above procedure pattern with stoichiometric ratios of 0.78 g of copper nitrate, 2.022 g of ferric nitrate and optimized volume of green fuel. The experimental preparation steps of synthesized of undoped CuFe_2_O_4_ (UCF) and LCF NMs as displayed in Fig. 1.

### Photo-catalytic dye degradation activity

The photo-catalytic dye decolouration activity of synthesized host and LCF NMs on FOR dye was carried out under Sunlight irradiation. This dye decolouration activity was conducted in between 10.30 AM and 2.30 PM, due to its high intensity of Sun-rays and also, prevents intensity fluctuations. In the reported research, 40 mg of synthesized of nanomaterial was processed with 250 mL of 20 ppm-FOR dye solution in a circular-glass reactor. During the experimental practices, 3 mL of FOR dye solution was collected at regular time interval until completion of dye-degradation and measure its adsorption by UV–Visible absorbance spectroscopy.

### Antibacterial activity

Antibacterial investigation of prepared LCF NMs is conducted using LB agar media by paper disc method. The media was poured into the sterilized petriplates in a quantity of around 25 mL and allowed to solidify. Using a plate spreader, 200 µL of each of the inoculums Bacillus subtillis (gram positive) and Pseudomonas aeruginosa (gram negative) were put into agar plates. Sterile discs (5 mm diameter) are plated on the plate with 10 µL of different concentrations of 100 µg, 200 µg and 300 µg test sample and positive control (Streptomycin and Ampicillin). The plates were then incubated at 37 °C for 24 h. The anti-microbial activity was assayed by measuring the diameter of the inhibition zone formed around the disc in millimeter.

### Instrumental characterizations

The structural variables of prepared nano-ferrites were investigated by (P-XRD) Shimadzu powder X-ray diffractometer (operating at 40 kV and 30 mA CuKa (λ = 1.541 Å) radiation with a nickel filter at a scan rate of 2° min^−1^). FT-IR examination of nano-ferrites were carreid out by a Shimadzu’s FT-IR spectrophotometer with KBr pellets at 400–4000 cm^−1^. The surface morphology of nano-ferrites were investigated by Scanning Electron Microscopy (SEM) (TESKON MIRA 3) (accelerating voltage up to 10 kV using Tungsten filament). The UV–Vis absorption spectral analysis was measured by Shimadzu UV–Vis spectrophotometer model 2600. Electrochemical studies of prepared nanomaterials were investigated by CHI608E potentiostat in 0.1 M KCL at different scan rates.

## Results and discussion

### P-XRD examination

**Figure 2:**
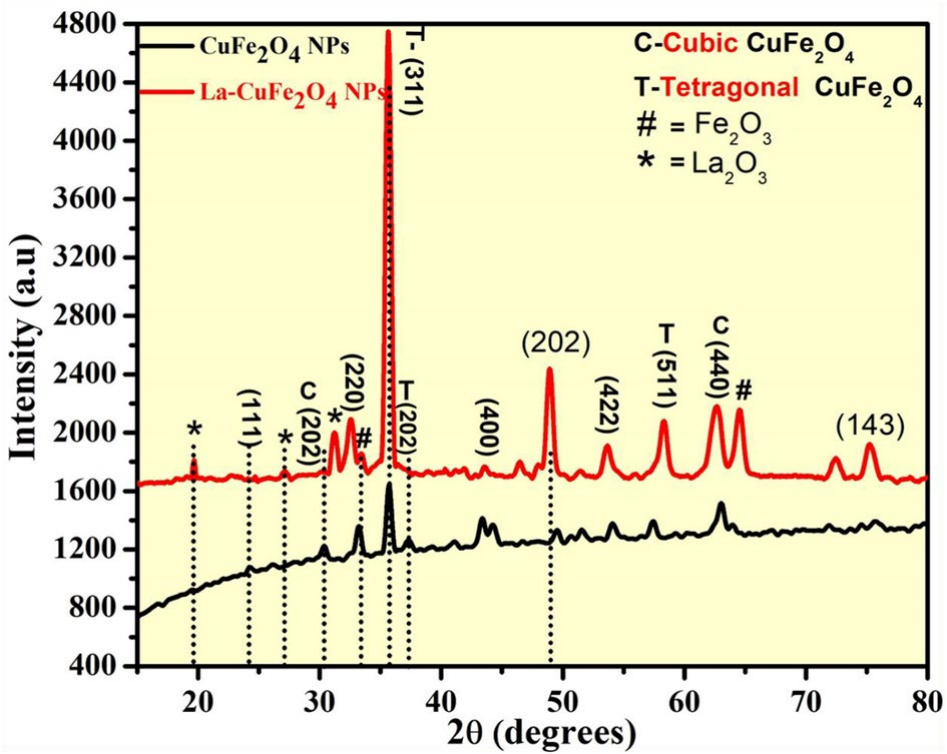
P-XRD spectral studies of UCF and LCF NMs.

The P-XRD investigation shows purity and existence of phases (cubic and tetragonal) of synthesized UCF and LCF NMs synthesized from combustion process using bio-fuel. These structural variables of prepared nano-doped-ferrites were examined through P-XRD spectral analysis recorded in the ranges of 10–80 nm at diffraction angle 2θ as depicted in Fig. 2. The appearance of diffraction peaks in P-XRD patterns of (111), (202), (220), (311), (400), (422), (511), (440) and (143) were well matched with standard JCPDS file No. 01-089-2531. These observed diffraction peaks can be indexed to cubic and tetragonal structures of synthesized nanoparticle^26–29^. The higher intensity peaks at 35.49, 48.96, 58.24, 62.77 and 64.24 corresponds to higher the crystallinity of synthesized doped nanoparticle. The crystallite sizes of achieved host and La doped CuFe_2_O_4_ nanoparticles were calculated via Scherrer’s expression [Eq. (1)] and its average crystallite sizes were reported to be 22–36 nm. The structural properties of prepared nanoparticles such as nanoparticle size, dislocation density (δ), stress, strain, stacking fault etc., are measured and reported in the Table 1^10,14^.

1 $$d=\frac{k\lambda }{\beta cos\theta }$$

2 $$ \delta = \frac{1}{{{\text{D}}^{2} }} $$

3 $$ {\text{SF}} = \left[ {\frac{{2\pi^{2} }}{{45\left( {3\tan \theta } \right)^{1/2} }}} \right] $$

**Table 1:** The various structural properties of prepared host and doped nanoparticles.

hkl	2θ	θ	β × 10^−3^	$D = \frac{0.9\lambda }{{\beta \cos \theta }}$	Strain ε = 10^−3^	Stress σ = ϵγ × 10^6^ m	$\delta = \frac{1}{{D^{2} }}10^{15}$	$SF = \frac{{2\pi^{2} }}{{45\left( {3\sin \theta } \right)^{1/2} }}$
311 host CuFe_2_O_4_	35.68	17.84	10.85	36.4	1.026	1.701	1.57	0.4508
311 La-CuFe_2_O_4_	35.60	17.8	10.8	22.4	1.532	2.12	0.748	0.2895

### Examination of morphological changes for synthesized nanomaterial

**Figure 3:**
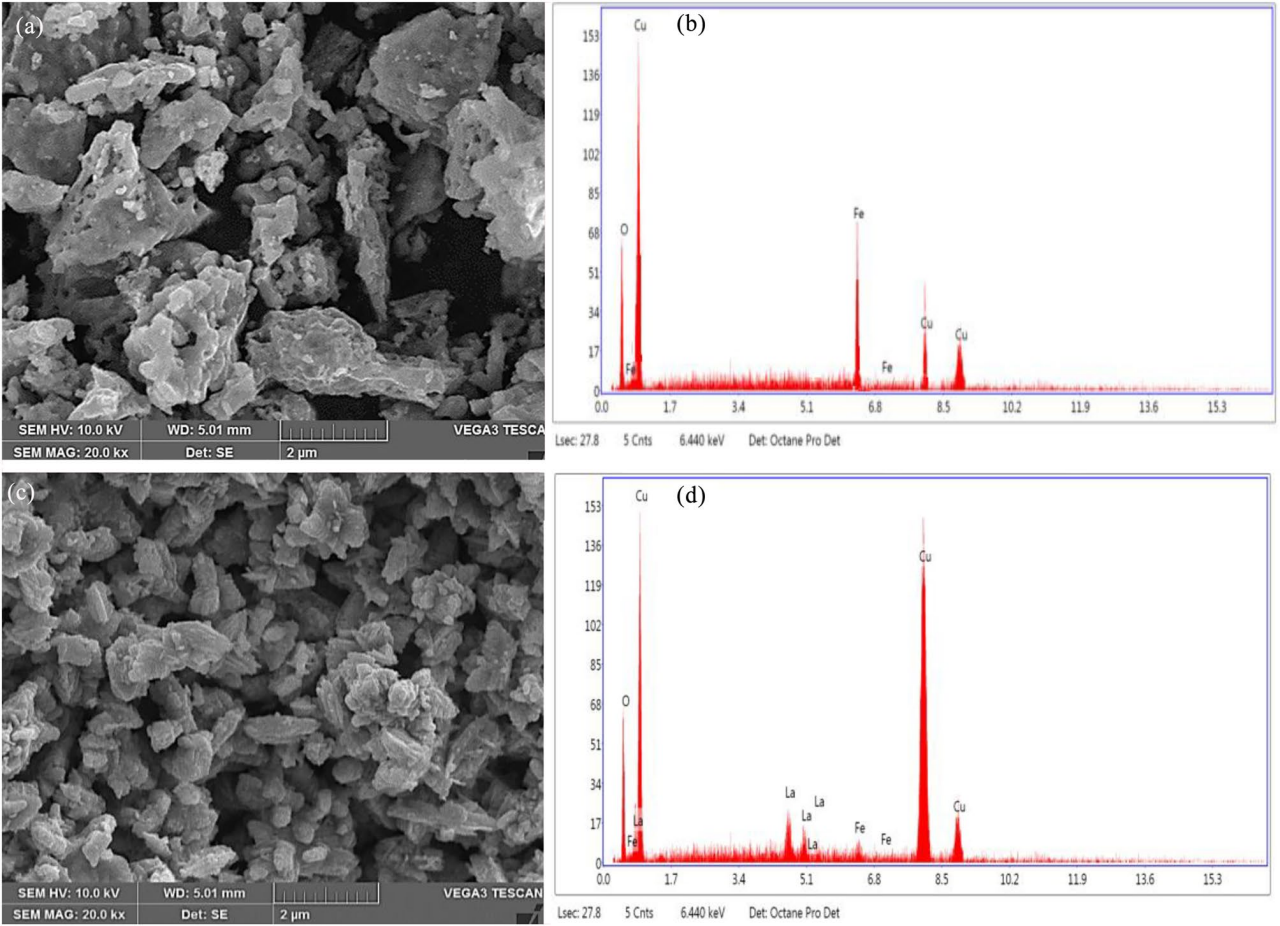
SEM-EDAX of (**a**, **c**) UCF NMs and (**b**, **d**) LCF (5 mol%) NMs.

SEM micrographs of UCF and LCF (5 mol%) NMs nanoparticles achieved from bio-extract assisted combustion process were as depicted in Fig. 3a and b respectively. In Fig. 3a, it can be observed that host nanoparticles possess well assembly of bundled nanomaterials obtained by aggregation of several atoms or nanoparticles and also, porous nature with appearance of small voids. The small changes in morphological structure like existence of flower like shapes with no viods and porous structures were observed for LCF (5 mol%) nanoparticle than that of UCF NMs (Fig. 3c). The existence of chemical constituents in synthesized materials were investigated by using EDAX studies. Figure 3b and d shows the EDAX analysis of UCF and LCF NMs respectively, confirms the existence of La^+^ ions in synthesized doped samples and its elemental compositions have been reported in Table 2.

**Table 2:** The elemental compositions analysis of synthesized samples.

Element	Host CuFe_2_O_4_	La doped CuFe_2_O_4_
	Atomic %	
OK	31.48	39.63
LaL	0.0	2.79
FeK	3.68	1.64
CuK	64.84	55.93

**Figure 4:**
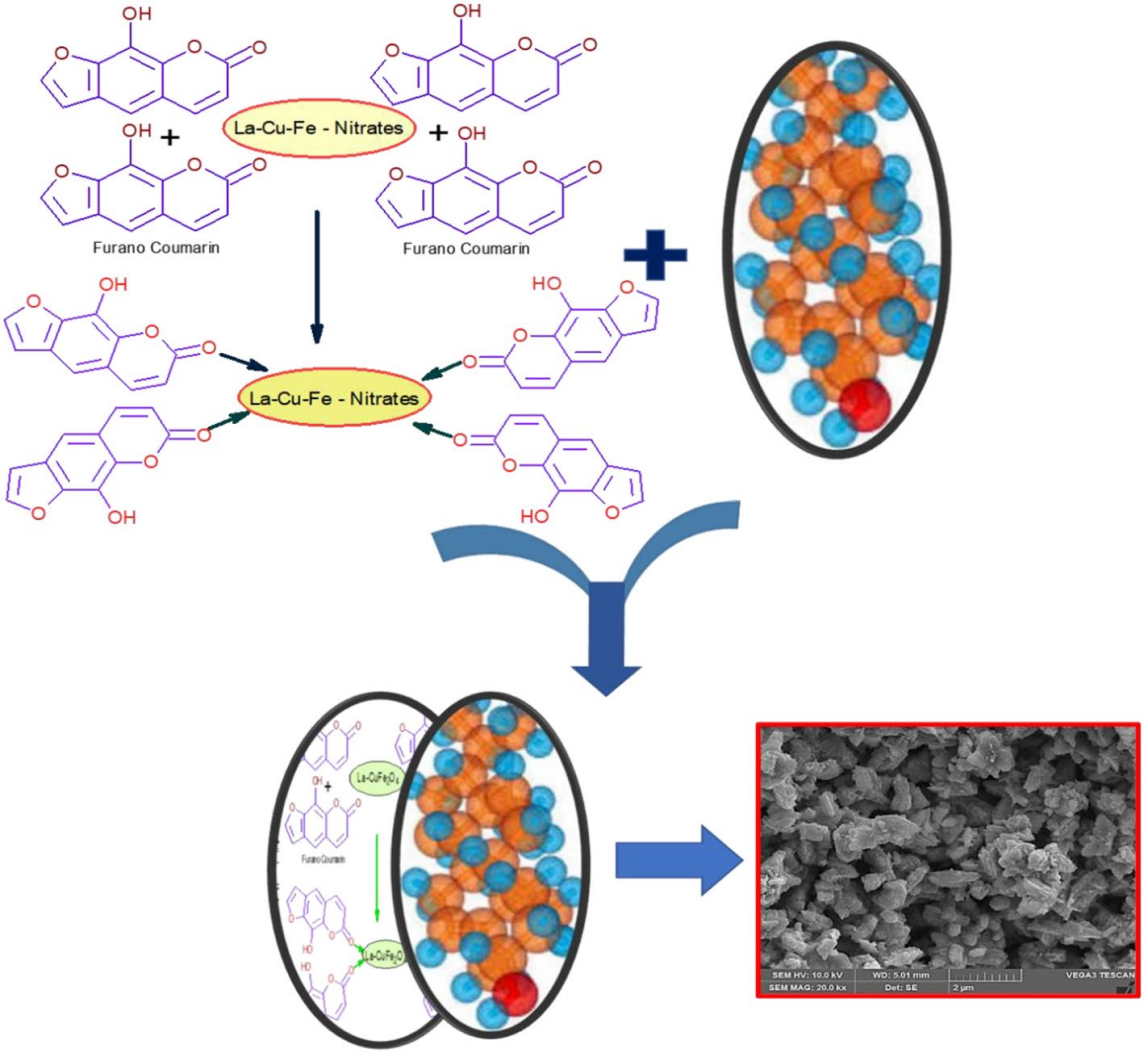
The presumptive schematic representation of coordination bond formation.

Generally, these morphological changes were noted for synthesized material from combustion process, which is due to the impact of presence of bio-fuel extract. These green extract contains bio-active substances such as xanthotoxol, coumarin, etc., shows vital role in morphological alterations during solution combustion process. These bio-active substances are involved in coordination bond formation with respective metal atoms (La^+^, Cu^2+^ and Fe^3+^) and lot of gaseous molecules with heat has been evaluated during combustion reaction and produces a spinel-ferrite nanostructured materials^20^. The presumptive schematic representation of coordination bond formation with respective metal atoms as displayed in Fig. 4.

**Figure 5:**
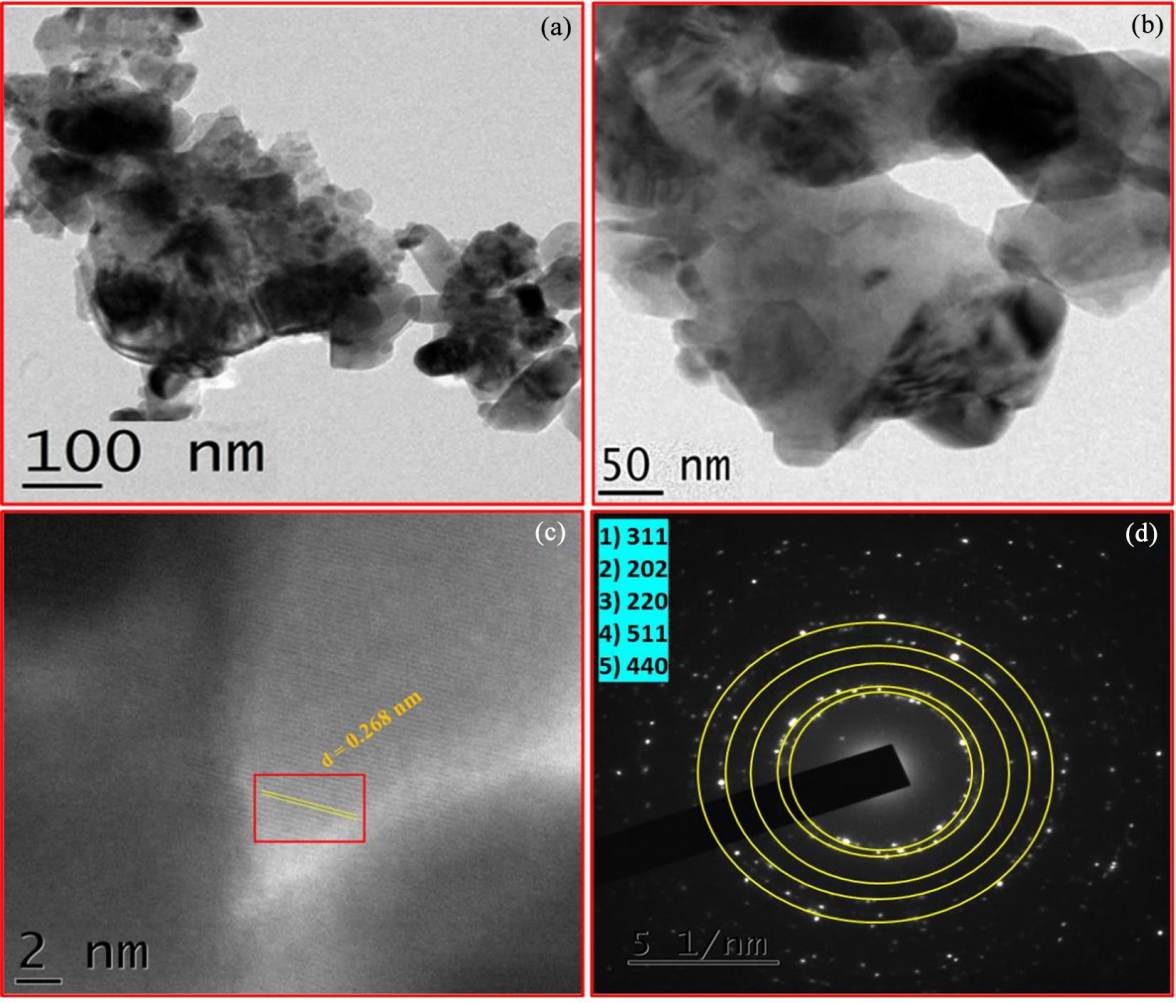
TEM images (**a**, **b**); HRTEM (**c**) and SAED pattern (**d**) of LCF (5 mol%) NMs.

Figure 5 represents TEM morphological analysis of prepared UCF and LCF (5 mol%) NMs. This TEM images clearly shows the assembly of smaller nanoparticles leads form an agglomerated structure with an increased surface area (Fig. 5a,b). The lattice spacing and interplanar distance of LCF (5 mol%) NMs was measured by HR-TEM analysis and its lattice-fringe was observed to be 0.268 nm, which related to the diffraction peak (311) of tetragonal phase (Fig. 5c). The occurrence of ring-pattern in Selected Area Electron Diffraction (SAED) indicates that higher crystallinity of LCF (5 mol%) NMs (Fig. 5d). These ring-patterns are well matches with the (311), (202), (220), (511) and (544) plane of La doped CuFe_2_O_4_ nanomaterial. The TEM investigation further provides an supporting analysis of the formation of LCF NMs and absence of other impurities.

### BET surface area examination Brunauer–Emett–Teller (BET)

**Figure 6:**
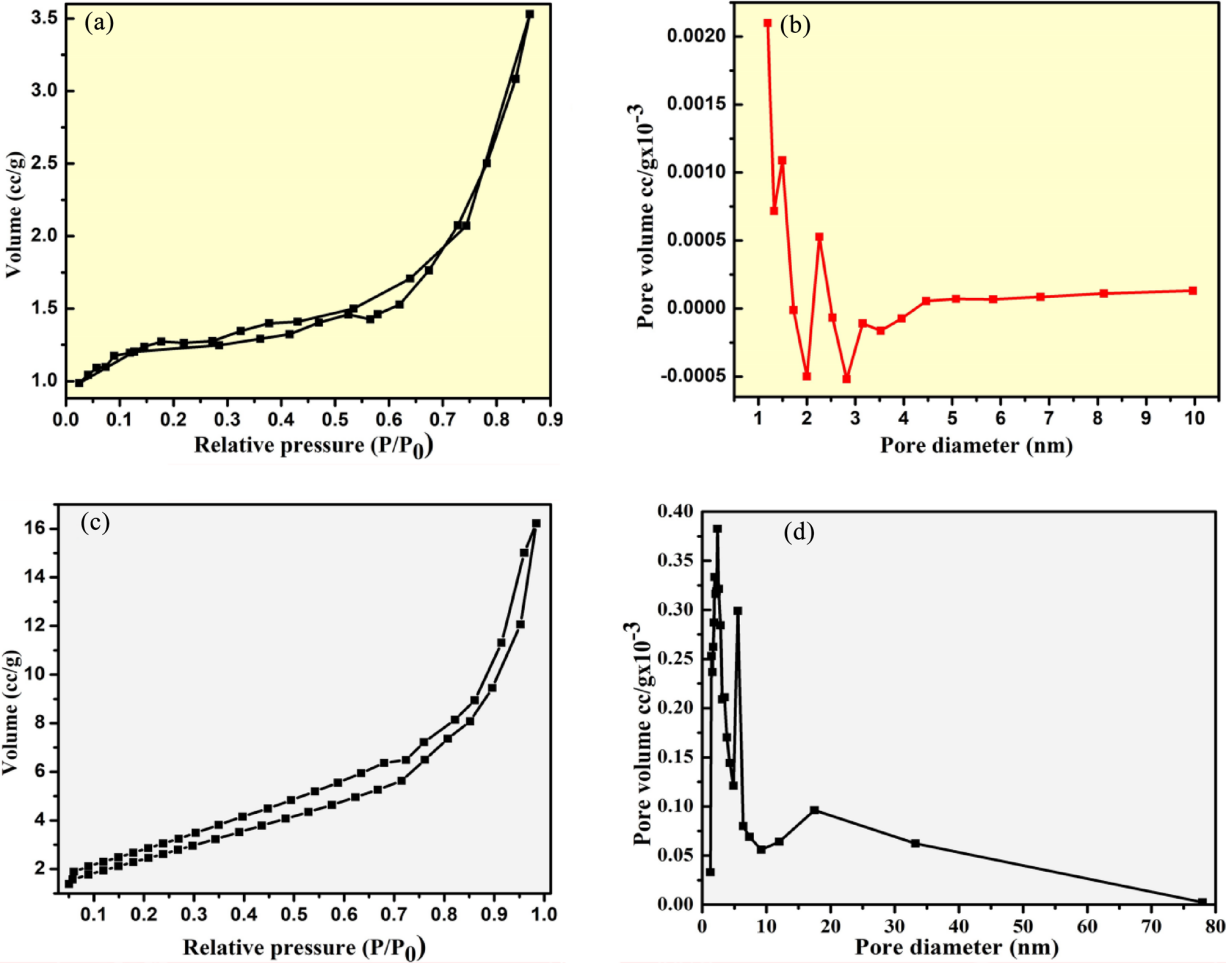
(**a**, **c**) The nitrogen adsorption–desorption isotherms; and (**b**, **d**) pore volume distribution graph of undoped and La doped copper-ferrite nanoparticle.

Figure 6a and c illustrates the typical specific surface and pore-diameter of synthesized host copper-ferrite and La^+^ ion doped copper-ferrite nanoparticles respectively via N_2_ adsorption–desorption isotherms performed using BET analysis. As per the literature, the synthesized nanoparticle from combustion route exhibits a higher surface area, which is due to production of heat (exothermicity)^30^. However, the synthesized La^+^ ion doped copper-ferrite nanoparticle having larger surface area than that of host copper-ferrite nanoparticle. The calculated BET analysis parameters of prepared materials such as surface area (S_BET_ [m^2^ g^−1^]), pore diameter (Dp [nm]) and pore volume (Dv [cm^3^ g^−1^]) were reported in Table 3. However, increasing specific surface area of La^+^ ion doped copper-ferrite nanoparticle (80.88 m^2^ g^−1^) is due to the uniform distribution of atoms/particles and presence of dopant fraction as observed from scanning electron microscopy and P-XRD investigations.

**Table 3:** The calculated BET analysis parameters of prepared materials.

Samples	Surface area, S_BET_ (m^2^ g^−1^)	Total pore volume (cm^3^ g^−1^)	Pore diameter (nm)
CuFe_2_O_4_	44.1	3.81	0.088
La-CuFe_2_O_4_	80.88	15.21	0.302

Thus, the obtained results of undoped and La^+^ ion doped copper-ferrite nanoparticle having an average pore-diameters of 0.088 and 3.02 nm respectively, which displaying the existence of mesopores nature of synthesized La^+^ ion doped copper-ferrite nanoparticle^10^. The pore-diameters of synthesized UCF and LCF NMs were noted by BJH adsorption-isotherms curves as displayed in Fig. 6b and d respectively. This examination shows the absences of pores in macropore domain (i.e., 500 Å) for synthesized doped copper-ferrite nanoparticle, which representing that the pores are exist in mesopore domain and thus, the synthesized doped-nanoparticle is mesoporous nature^31^. Therefore, the obtained results of different parameters from BET investigations confirms that LCF NMs possesses excellent surface area with porosity, which is directly corresponds to the higher photo-catalytic activities.

### FT-IR examination

**Figure 7:**
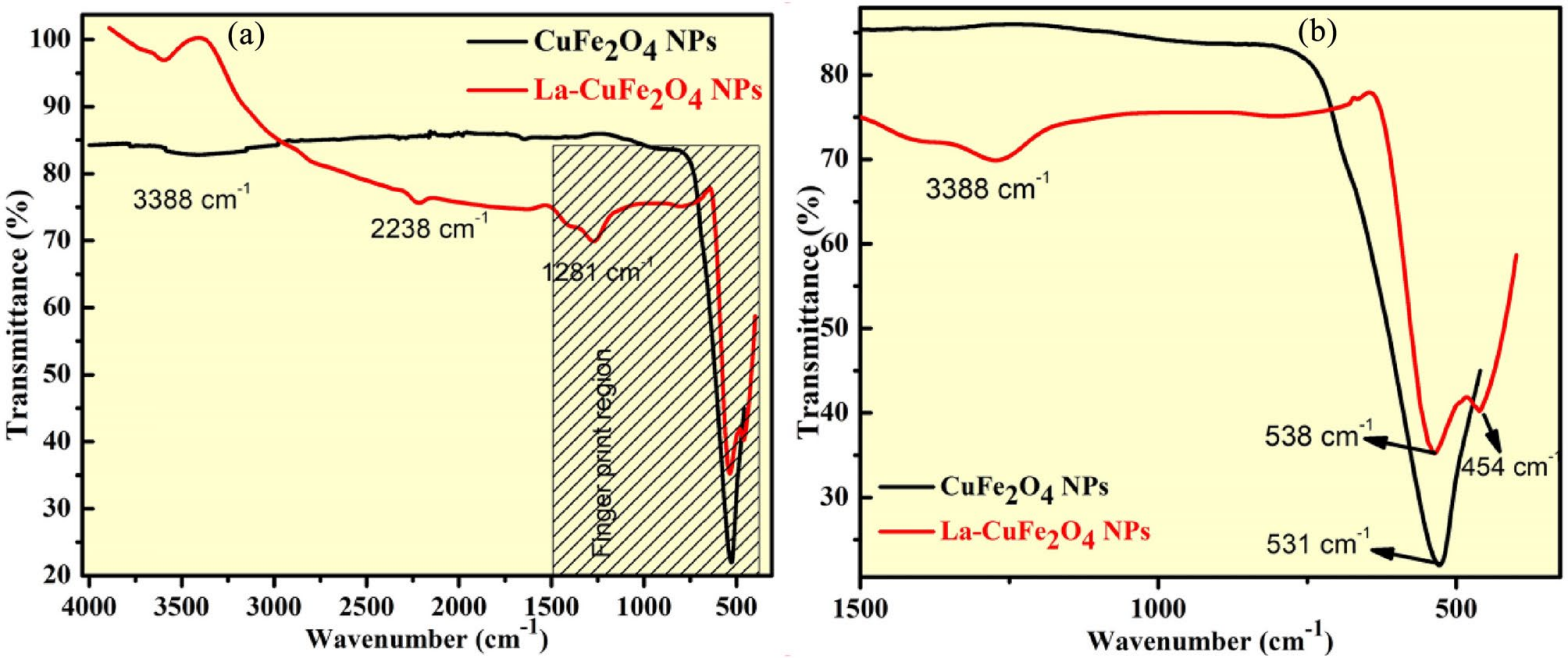
(**a**) FT-IR spectral studies and (**b**) Enlarged portions of FT-IR spectral studies for synthesized UCF and LCF NMs.

FT-IR spectral studies of synthesized UCF and LCF NMs shows the nature of bonding with formation of metal–oxygen (M–O) vibrations in octahedral (OS) and tetrahedral sites (TS) as depicted in Fig. 7a. The obtained FT-IR spectral outcomes validates presence of functional linkages associated with La-Cu ferrite noticing corresponding absorption bands at 454 cm^−1^ and 538 cm^−1^ (Fig. 7b). These characteristic functional linkages noticed in these wavenumbers reveals existence of spinel phase structure by consuming two sub-lattices of OS and TS^32,33^. Thus, the presence of absorption peaks in finger print region at higher region of 538 cm^−1^, indicating intrinsic stretching vibrations of metal at TS and band at lower region of 454 cm^−1^ is corresponding to OS metal-stretching^34^. The presence of broader band at 3388 cm^−1^ and lower peak 2238 cm^−1^ corresponding to the stretching vibrations of hydroxyl ions absorbed on synthesized photocatalyst surfaces and carbon-hydrogen stretching vibrations respectively. Thus, FT-IR spectral studies of synthesized nanoparticles specifically assist the formation of La–Cu-ferrite as reported in P-XRD examination.

### Optical investigations

**Figure 8:**
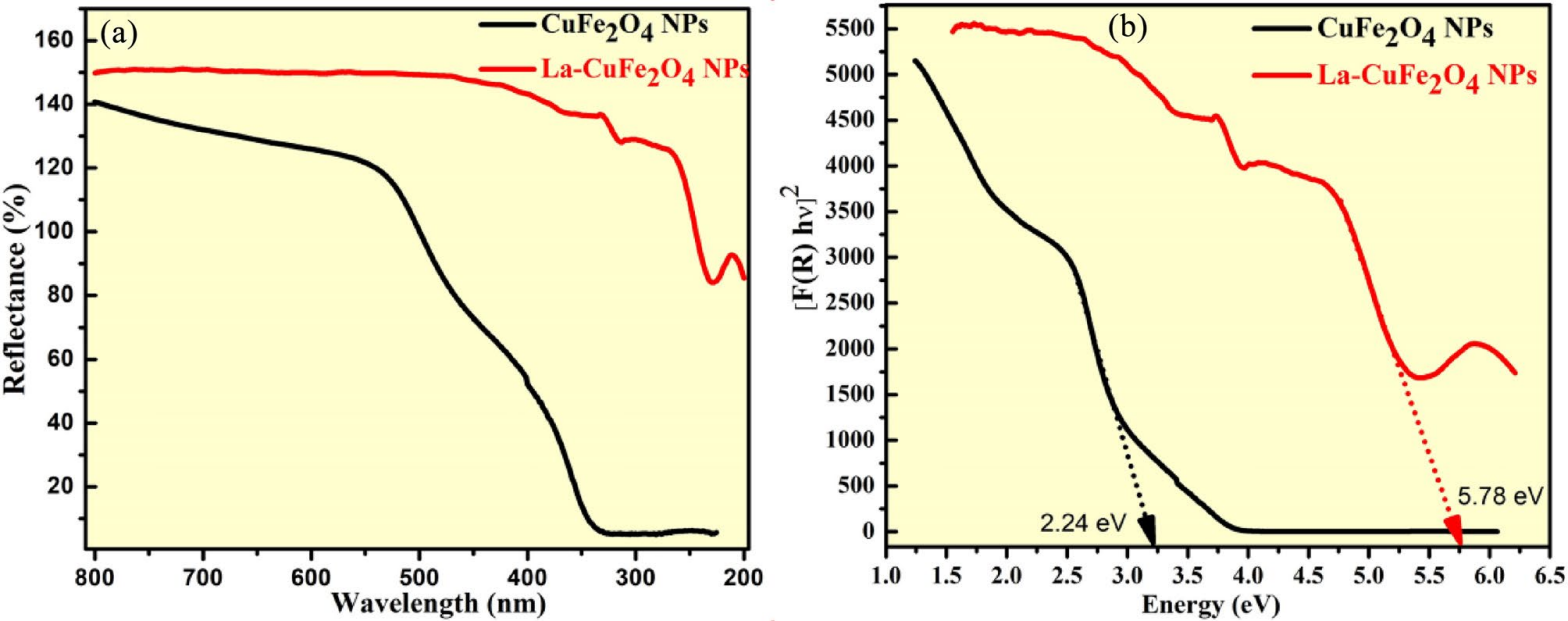
UV–Vis reflectance spectral studies of prepared UCF and LCF NMs.

The energy band-gap examinations of prepared UCF and LCF NMs were measured by Tauc plot as depicted in Fig. 8. This optical parameter of prepared nanoparticles were recorded by DRS spectral technique ranges between 200 and 800 nm (Fig. 8a). The diffuse reflectance of prepared UCF and LCF NMs (R), is directly related with absorption coefficient (*K*) and inversely proportional to scattering coefficient (*S*) are measured by the Kubelka–Munk method^35^.

4 $$F\left(R\right)=\frac{{(1-R)}^{n}}{2R}=\frac{K}{S}$$

The Tauc equation and linear absorption coefficient (α) equations are used to determine the energy band-gap (Eg) of prepared UCF and LCF NMs from following relations;

5 $$\alpha =\frac{{{C}_{1}(h\nu -Eg)}^\frac{1}{2}}{h\nu }$$

6 $${[F\left(R\right)h\nu ]}^{2}={C}_{2}\left[h\nu -Eg\right]$$

The band-gap of prepared UCF and LCF NMs were observed to be 2.24 and 5.78 eV respectively by plotting [F(R)hν]^2^ v/s hν as shown in Fig. 8b. The wider band-gap energy of doped CuFe_2_O_4_ Nps due to incorporation of La ions in crystal lattice of nanomaterial, which is connected to higher photo-degradation action than that of host material.

### Electrochemical studies of LCF NMs

**Figure 9:**
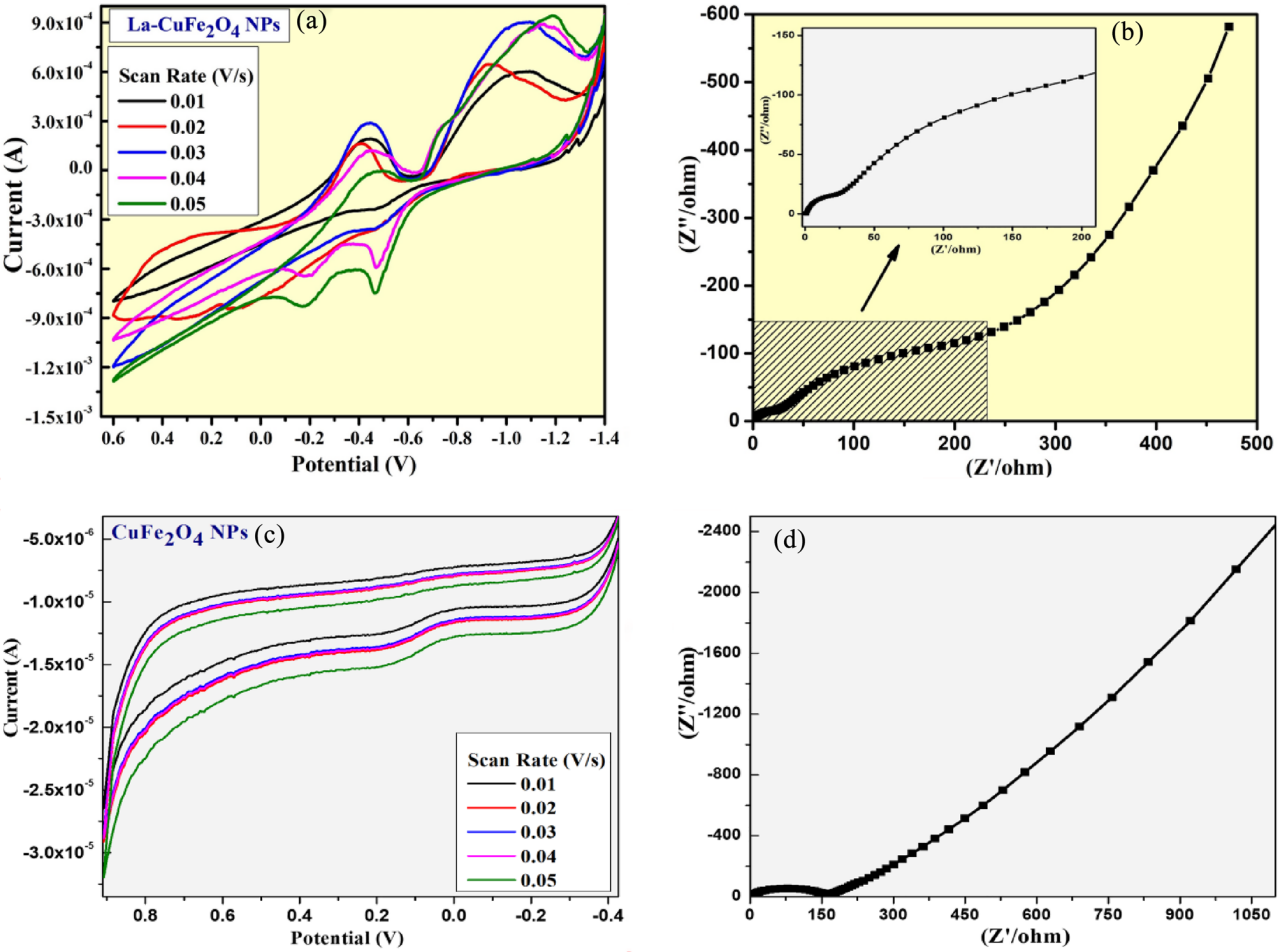
(**a**, **c**) CV plots of UCG and LCG electrodes; (**b**, **d**) Nyquist plots of UCG and LCG electrodes in at the scan rate of 0.01–0.05 V/s in 0.1 M KCl respectively.

The systematic measurements of electrochemical behaviour for prepared UCF and LCF NMs by electrochemical-work station (potentiostat) in 3-electrode arrangement with 0.1N KCl electrolyte as presented in Fig. 9. The potentiostat analysis is most significant to understand electrochemical practices of achieved material that leads to give a redox reaction under suitable electrolyte carried out by cyclic voltammetry (CV) and impedance spectroscopy (EIS) investigations. Thus, the synthesized LCF NMs was successfully explored for its efficient activities towards redox potentials, supercapacitor behaviour (charging-discharging nature) and sensor studies carried out by electrochemical investigations. The CV investigations of resulted Undoped-CuFe_2_O_4_ (UCG) and La doped CuFe_2_O_4_-Graphite (LCG) electrodes demonstrates the effective capacitance performance due to current generation from electron transfer between redox species and electrodes (Fig. 9a,c). As displayed in Fig. 9a and c, the CV plots of synthesized UCG and LCG were performed in the potential ranges of (+ 0.9 to − 0.4 V) and (+ 0.6 to − 1.4 V) under different scan rates of 10 to 50 m V/s respectively.

The specific-capacitance values of prepared materials were achieved from below specified theory [Eq. (7)], which supports that the LCG electrode has superior specific-capacitance (135 F/g) than those of UCG electrode (48.8 F/g). The doping of La metal into host CuFe_2_O_4_ nanomaterial displayed a rectangular shape with variations in redox peak potential curve due to the impact of redox reactions, this specifies an electrical double-layered charge mechanism. The specific-capacitance of synthesized UCG and LCG electrodes were recorded by the influence of encircled curves in achieved CV graphs by means of its equation mentioned below [Eq. (7)]

7 $${C}_{SP} = \frac{\int IdV}{v*m*\Delta V}$$

where; the specific-capacitance determined from CV is C_SP_; weight of sample (F/g) as m; current (A) as I, v = scan-rate (mV/s) and ΔV = potential windows (V).

EIS analysis illustrates the supercapacitor behaviour of modified UCG and LCG-electrodes by the influence of its semi-circle arc appeared as presented in Fig. 9b and d. These arc is observed at higher frequency region and vertical-line in lower frequency region corresponding to the superior capacitance of LCG (26 Ω) than those of CG (175 Ω) nanomaterial. As a result, higher capacitance of sample shows less charge-resistance transfer and larger the ratios of charge migration in between electrode and electrolyte.

**Figure 10:**
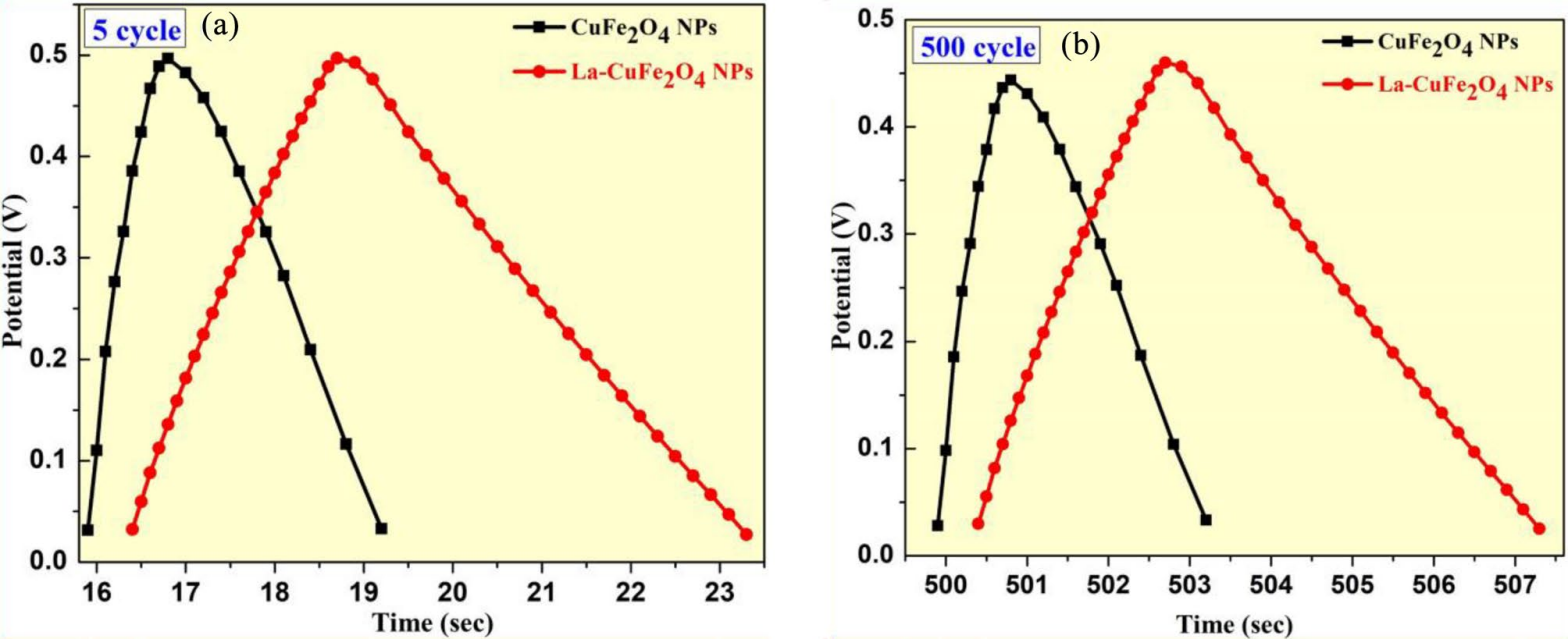
(**a**, **b**) The charging and discharging plots of synthesized UCG and LCG electrodes.

The charging and discharging behaviour of synthesized UCG and LCG electrodes were examined at 0.5 A/g current density as shown in Fig. 10. This achieved results shows well concordance with pseudo-capacitive behaviour of linear-triangular configurations. The specific capacitance values of UCG and LCG electrodes mentioned above were ascertained by the impact of charging-discharging curves. Hence, this studies are systemically investigated for synthesized samples over a 2000 charging-discharging cycles and carrying almost same capacitance stability around 93% (Fig. 10a,b). Therefore, the observed data confirms that the LCG material has very good cycle-life and cyclic stability than those of UCG material.

### Sensor examination of LCG electrode

**Figure 11:**
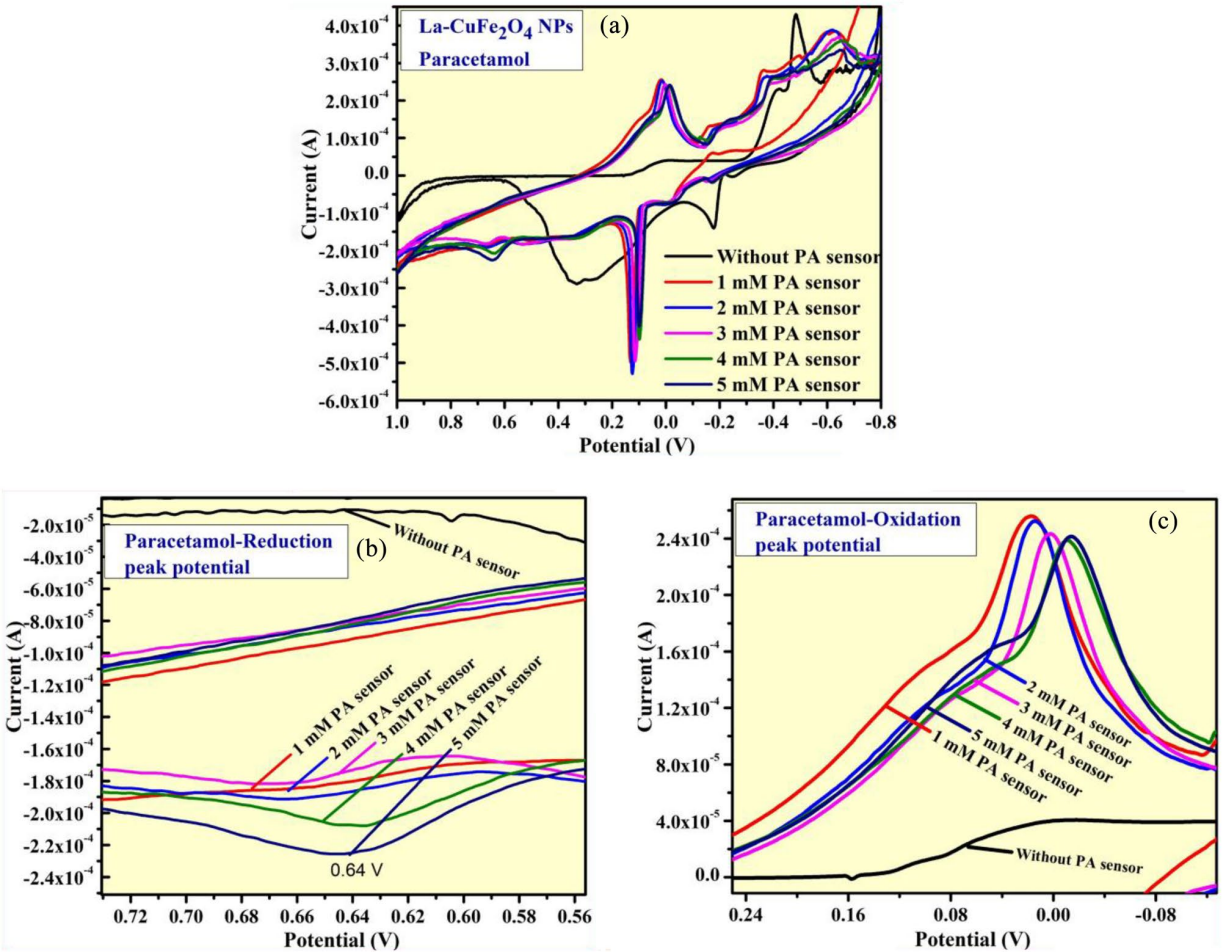
(**a**) CV analysis of LCG electrode for paracetamol detection; (**b**, **c**) The enlarged portions of reduction and oxidation curve peaks at 1–5 mM in 0.1 M KCl.

The electrochemical sensor investigation of synthesized LCG electrode was performed in the potential range of + 1.0 to − 0.8 V under different scan rates of 10 to 50 m V/s. Thus, the prepared LCG-electrode is effectively shows potential sensing activities towards heavy metal (Lead (Pb) content) chemical and paracetamol drug molecule examined by CV studies in 0.1 N KCl medium. Thus, the sensing investigation of of UCG electrode was carried out by CV analysis using different concentrations (1–5 mM) of paracetamol drug at potential value ranges from + 1.0 V to − 0.8 V as displayed in Fig. 11a. The maximum intensity of reduction and oxidation-peak potentials observed at + 0.64 V and − 0.012 V respectively, representing the paracetamol content present in 0.1NKCl electrolyte as depicted in Fig. 11b and c. The electrochemical activity of these studies is compared with other reported studies as given in Table 4.

**Table 4:** Comparison of electrochemical studies of different nanomaterials for various sensors.

Sample	Electrochemical studies (sensor detections)	Scan rates	Reference
ZnO NPs	Paracetamol sensing properties	0.03 V/s	^36^
ZnFe_2_O_4_ NPs	Mifepristone and Misoprostol (1–5 mM) chemical	0.03 V/s	^37^
ZnFe_2_O_4_:Cu NPs	Mifepristone–misoprostol (1–6 mM) chemical	0.03 V/s	^38^
Clay/MgO nanocomposite	Stannous chloride sensor, dextrose, and eye drop chemicals	0.03 V/s	^39^
MgNb_2_O_6_:Sm3^+^	Paracetamol and alcohol	0.03 V/s	^40^
Nano ferrite bentonite clay	Dextrose biomolecule	0.03 V/s	^41^
ZrO_2_:Mg^2+^	Paracetamol sensor and eye drops	0.03 V/s	^46^
CuFe_2_O_4_ NPs	Specific capacitance	1–5 mV/s	^20^
CuFe_2_O_4_: La^3+^	Paracetamol and lead sensor	0.03 V/s	Present work

**Figure 12:**
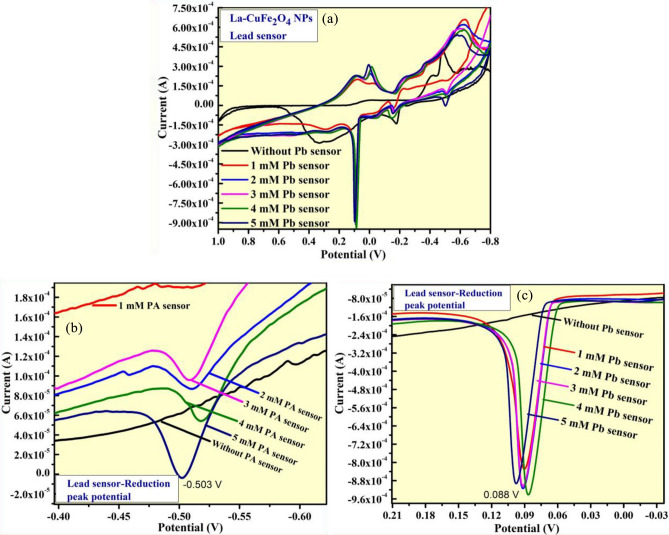
(**a**) CV analysis of LCG electrode for lead detection; (**b**, **c**) The enlarged portions of reduction curve peaks at 1–5 mM in 0.1 M KCl.

Further, the excellent sensing activity of prepared LCG electrode was observed for Lead-chemical content by CV analysis using different concentrations (1–5 mM) of lead nitrate solution at same potential value ranges mentioned above as displayed in Fig. 12a. the very high intensity of reduction potential peaks were recorded at + 0.88 V and − 0.503 V, confirms existence of lead content in used 0.1NKCl electrolyte with different concentrations (1–5 mM) of lead nitrate solution as displayed in Fig. 12b and c respectively^42–46^.

### Stability of LCG NMs

**Figure 13:**
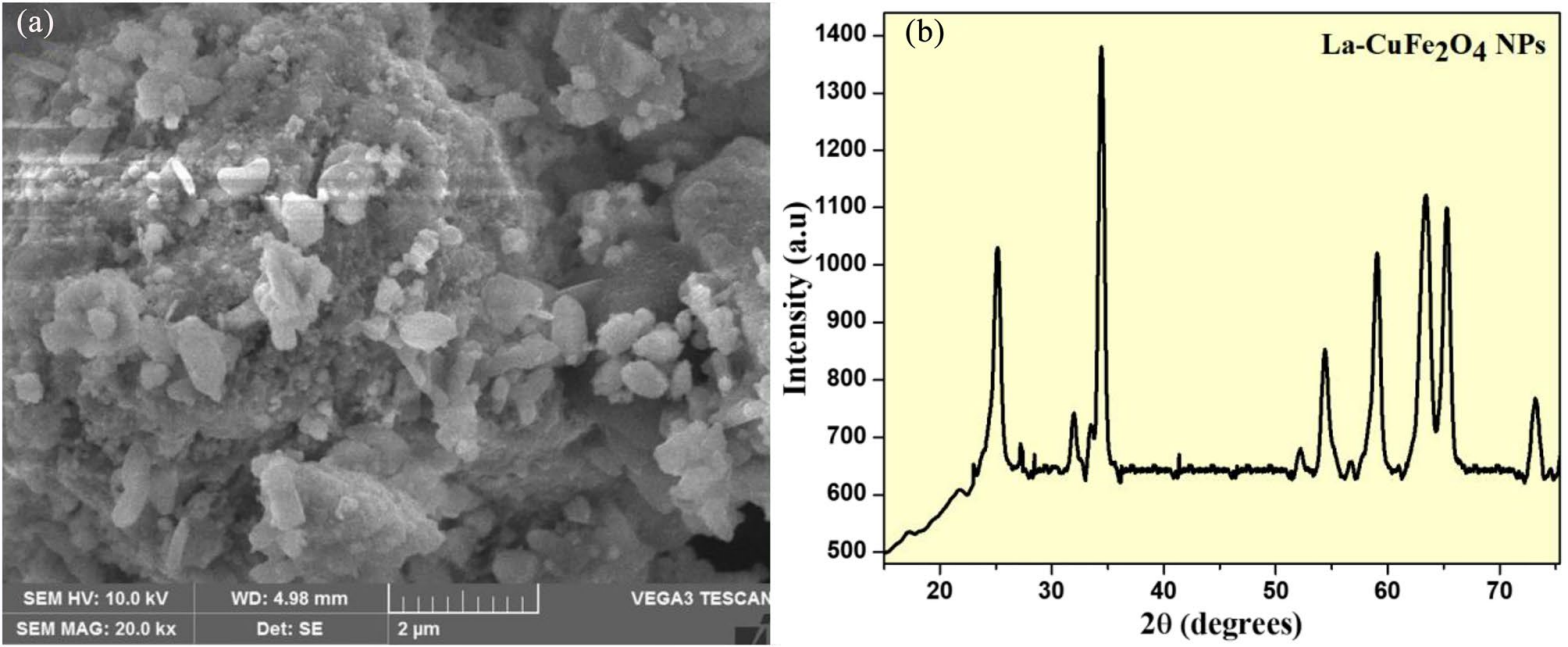
(**a**) SEM analysis and (**b**) PXRD studies of LCG electrode after the after long-time cycling.

The stability of the used LCG electrode for detection of paracetamol and Lead-chemical contents by CV analysis using different concentrations discussed above was confirmed by performing its characterizations by SEM morphological and PXRD analysis as shown in Fig. 13a and b respectively. SEM images shows the slightly changes in morphological structure than those of before performing the experiment. Also, the PXRD structural analysis showed an slightly shifting of diffraction peaks with appearance of additional peaks due to the graphite combination during preparation of LCF electrode. These results confirmed that the prepared LCF electrode with graphite has very good stability towards long-time cycling.

### Photocatalytic dye-degradation studies

The large quantity of toxic pollutants that was discharged into the water from various industrial-sectors causing serious ecological issues throughout the universe. In order to take off dye-contaminated water, the photocatalytic degradation route is a most significant method that has recently attracted greater attention towards removal of toxic pollutants in waste water. The heterogeneous nano-catalyst and organic dye solutions are present in different phases during this photocatalysis reaction^47,48^. The photo-dye-degradation route includes various type of reactions such as light absorption on surface of photocatalysts, radicals generations, dye decomposition and contaminants elimination by light oxidation activity. Due to their extensive range of electronic states and band energies, semiconductors are the most widely used heterogeneous photocatalysts because they possess intrinsic physico-chemical properties that distinguish them from metals and prevent the electron hole recombination caused by photo-activation. The filled valence band to the empty conduction band are also included in the band gap^49^. The photodegradation analysis of different photocatalyst for various dye as shown Table 5.

**Table 5:** Comparison of degradation efficiency of different photocatalyst for various dye.

Sample	Dyes	Degradation efficiency (%)	Time	Reference
BaTiO_3_/GO	MB	95%	3 h	^50^
SnS NPs (cubic and orthorhombic)	MB	88% and 98% respectively	120 min	^51^
SnS-nanocatalyst	MG	98%	75 min	^42^
ZnO	MB	98%	120 min	^36^
ZnFe_2_O_4_ NPs		98%		^37^
ZnFe_2_O_4_ :Cu NPs	Rh-B	94%	90 min	^38^
ZrO_2_ and ZrO_2_:Mg^2+^	Acid Green (AG)	74.46% and 88% respectively	105 min	^46^
CuFe_2_O_4_ NPs	MG	87.5%	140 min	^20^
Clay/MgO nanocomposite	Rh-B	90%	100 min	^39^
CuFe_2_O_4_	FOR	82.3%	90 min	Present Work
CuFe_2_O_4_: La^3+^		91.7%		

**Figure 14:**
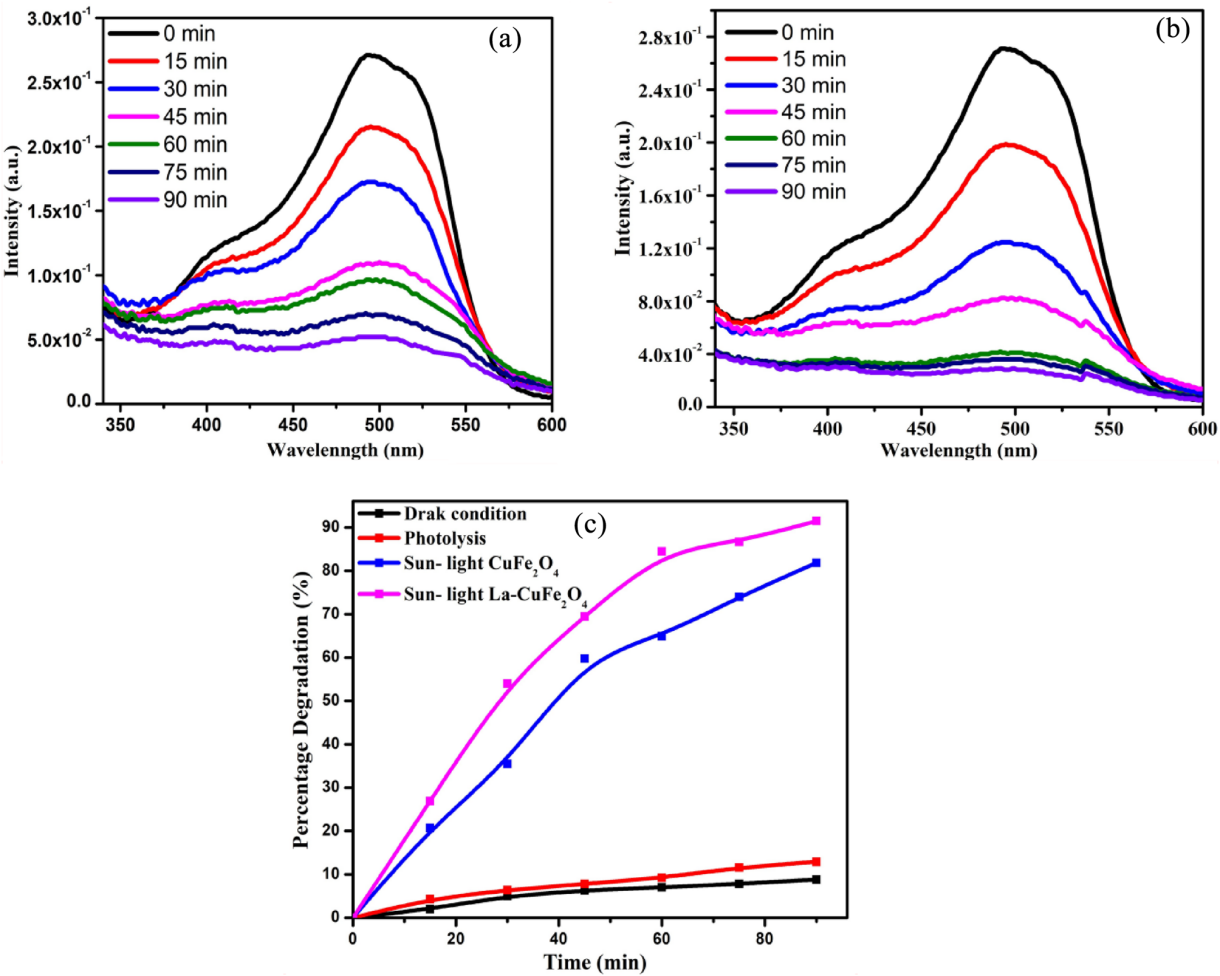
The occurrence in absorbance of FOR dye (20 ppm) in presence of synthesized (**a**) UCF NMs and (**b**) LCF NMs under Sun-light irradiation and (**c**) Photo-degradation percentage of FOR dye (20 ppm) in presence of synthesized materials under Sunlight-irradiation for 90 min.

The photocatalytic degradation activity of synthesized UCF and LCF NMs from were examined on FOR dye by the impact of Sun-light irradiation as displayed in Fig. 14a and b respectively. The heterogeneous photo-catalytic process on dye-decolouration reaction was performed with 20 ppm FOR dye solution and 40 mg of synthesized samples at 90 min under Sun-light irradiation. The absorbance of each degraded solutions for every 15 min were measured by UV–Visible absorbance spectroscopy (Fig. 14a,b). As a result, the LCF NMs has shown excellent photo-degradation performance on FOR dye than those of UCF NMs under Sun-light irradiation. The percentage dye-degradation performances of UCF and LCF NMs were measured and reported to be 82.3% and 91.7% respectively at 90 min under Sun light as shown in Fig. 14c. Additionally, the photo-degradation performance on FOR dye under dark and photolysis was noted to be 8.9 and 13.6% respectively (Fig. 14c).

**Figure 15:**
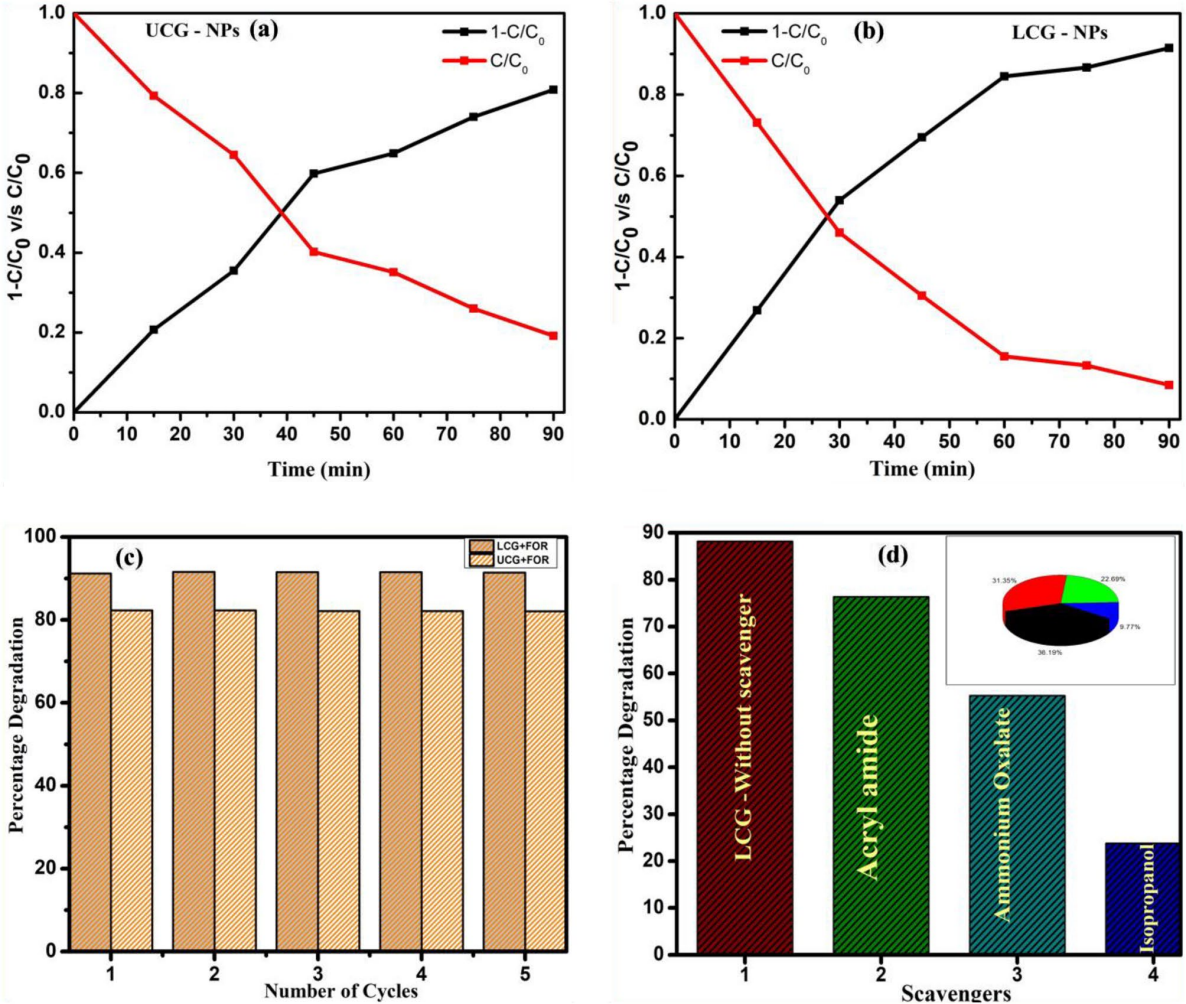
(**a**, **b**) The half life photo-degradation percentage of UCF and LCF NMs on FOR dye under Sunlight irradiation; (**c**) Re-usability investigation of synthesized photocatalyst for 5 consecutive recycle runs and (**d**) scavengers studies.

Further, the half time dye-decomposition examinations of UCF and LCF NMs on FOR dye under Sunlight irradiation were observed to be 38.5 and 26.7 min respectively through plotting (C/C0) v/s time as shown in Fig. 15a and b. This analysis confirms that the LCF NMs has greater dye-decomposition in its half of degradation time than those of UCF NMs. The recycle performance ability of LCF NMs on FOR dye decoloration was investigated for after completion of every cycle under similar conditions over 5 cycles as shown in Fig. 15c. Additionally, the photo-degradation performance on FOR dye measurements were supported by scavenging test under some scavengers as represented in Fig. 15d. The experimental photocatalytic degradation analysis was carried out with acryl amide, ammonium oxalate and isopropanol scavengers show 88.4, 53.6 and 25.6% respectively (Fig. 15d). This studies confirms that, isopropanol scavenger shows vital role in photocatalytic activity by blocking the holes. Hence, photo-degradation performance of FOR dye was successfully achieved by holes as an effective tool under Sun-light irradiation^33,34^.

**Figure 16:**
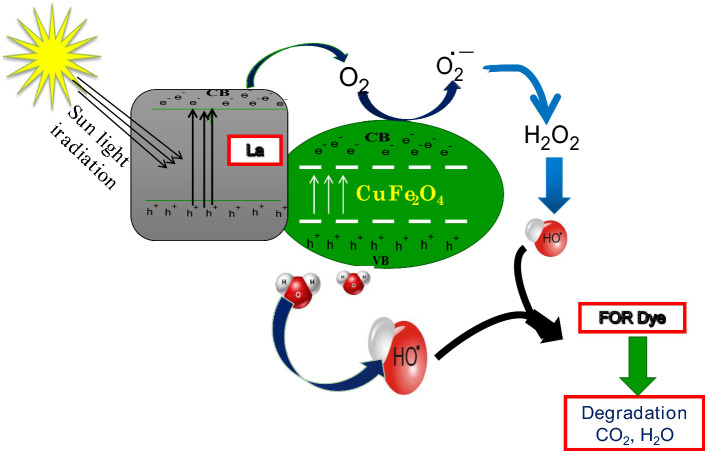
The probable photo-catalytic mechanism for degradation of FOR dye in presence of photo-catalyst under sunlight irradiation.

The red appearance of dyes is caused by chromophore groups and azo linkages (-N = N-), which exhibit a prominent peak at 496.6 nm wavelength due to the n → π* transition that is photo-catalytically degraded under sun-light radiation. At room temperature (RT), the UV–Visible absorption spectral analysis of FOR dye solution with prepared photocatalysts were captured from 0 to 90 min. These photocatalysts are get excited by capturing light radiation [Eq. (8)] and produces lot of ${h}^{+}$ (holes) and ${e}^{-}$(electrons) [Eq. (9)]. These charges are mainly responsible for the generation of radicals such as superoxide-radicals (${\mathrm{O}}_{2}^{\cdot -}$) by impact of electrons in conduction band [Eq. (10)] and hydroxyl radicals ${(OH}^{\cdot -})$ [Eq. (11)] by ${h}^{+}$ in valence band. More ${e}^{-}, {h}^{+}$ produced and effective charge transfer towards the FOR dye contribute to the ultra-fast dye degradation in presences of prepared photocatalysts and its possible mechanism as displayed in Fig. 16.

8 $${\mathrm{LaCuFe}}_{2}{\mathrm{O}}_{4}\mathrm{ NPs} +\left(Sun Light energy\right)---\to {\mathrm{LaCuFe}}_{2}{\mathrm{O}}_{4}\mathrm{ NPs}*\left(Energy\right)$$

9 $${\mathrm{LaCuFe}}_{2}{\mathrm{O}}_{4}\mathrm{ NPs}*\left(Energy\right)---- - \to {\mathrm{LaCuFe}}_{2}{\mathrm{O}}_{4}\mathrm{ NPs }\left({h}^{+}+{e}^{-}\right)$$

10 $${\mathrm{LaCuFe}}_{2}{\mathrm{O}}_{4}\mathrm{ NPs }\left({\mathrm{e}}^{-}\right)+{\mathrm{O}}_{2}----\to {O}^{2\cdot -}\left(Superoxide radical\right)$$

11 $${O}^{2\cdot -}+{H}_{2}O--------------\to {OH}^{\cdot }+{OH}^{-}$$

### Antibacterial studies and its mechanism

**Figure 17:**
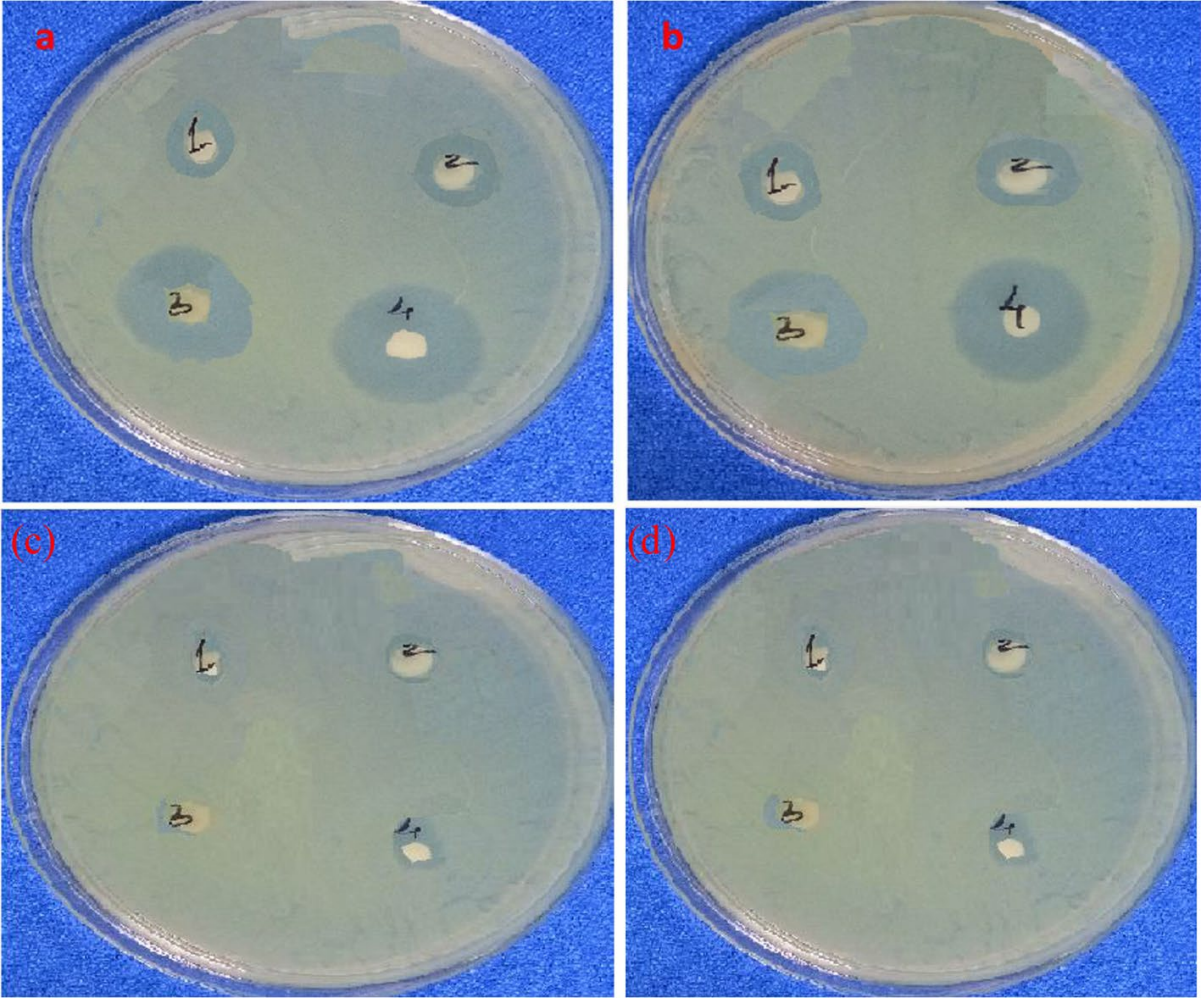
Inhibition zone of (**a**) *Bacillus subtillis (Gram positive) with* Streptomycin as positive control (4) (**b**) *Pseudomonas aeruginosa (Gram negative) with ampicillin* as positive control (4) for LaCuFe_2_SO_4_ sample of (1) 100 µg/mL (2) 200 µg/mL (3) 300 µg/mL concentrations and for host-CuFe_2_SO_4_ sample of (1) 100 µg/mL (2) 200 µg/mL (3) 300 µg/mL concentrations.

The antibacterial activity of the synthesized UCF and LCF NMs were investigated against various strains of gram positive and gram negative bacteria, such as *Bacillus subtillis* and *Pseudomonas aeruginosa* respectively. The bactericidal action of the sample was measured with respect to the zone of inhibition as shown in Fig. 17 and tabulated Table 6. Our research indicates that the tested LCF sample has very good activity than those of UCF NMs. Specifically, the LCF sample had considerable effect against both the bacteria's while the compound shown marginally high activity towards gram positive bacteria with an average inhibition zone of 19 mm.

**Table 6:** Zone of Inhibition in mm of LaCuFe_2_O_4_ compound against Bacillus subtillis and Pseudomonas aeruginosa.

Concentrations (µg/mL)	LaCuFe_2_O_4_		CuFe_2_O_4_	
	*B. subtillis*	*P. aeruginosa*	*B. subtillis*	*P. aeruginosa*
100	15	13	15	0.75
200	18	15	18	2.8
300	22	18	22	1.2
Control	26	25	26	10.5

**Figure 18:**
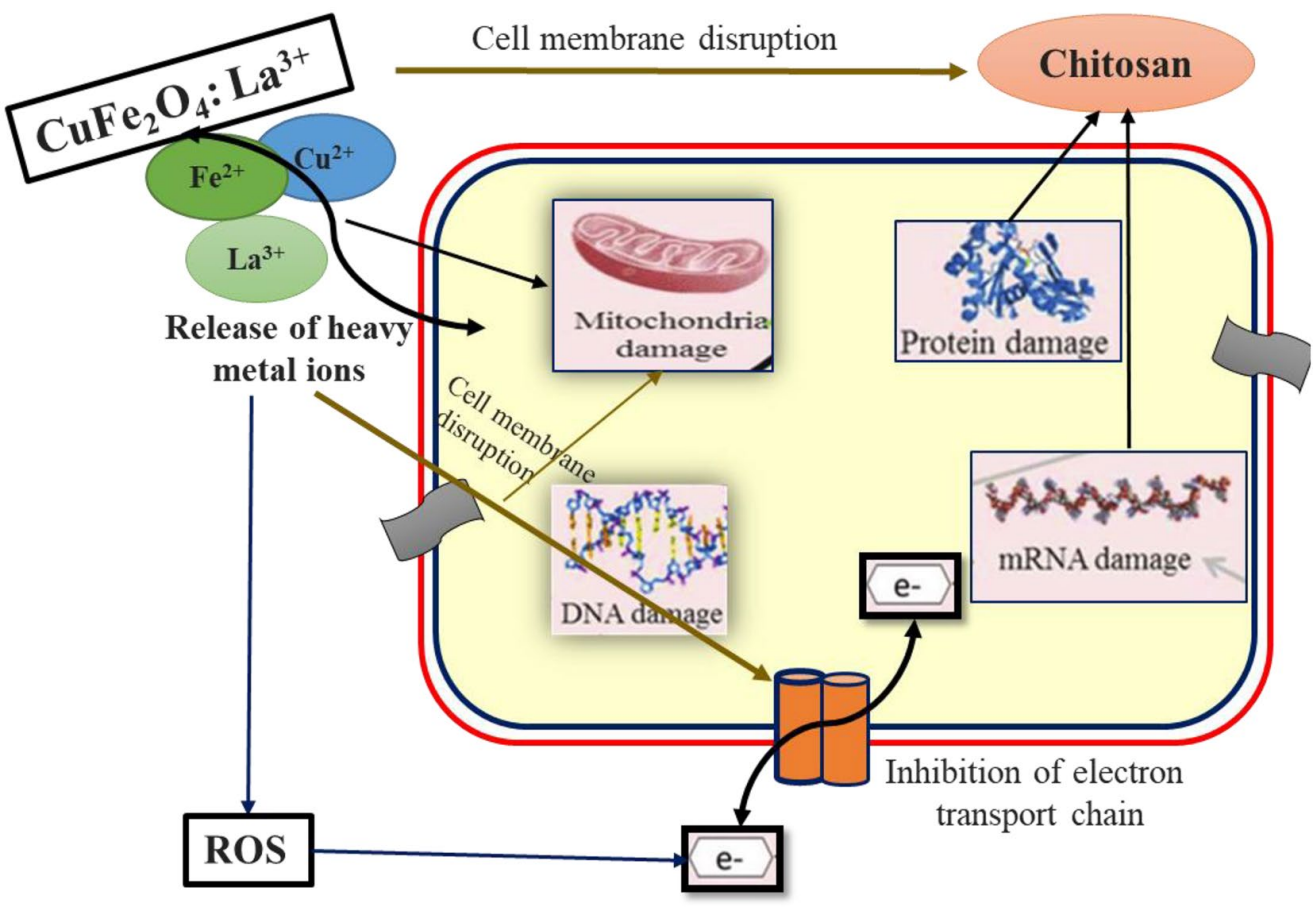
Probable mechanism for enhanced antibacterial activity of CuFe_2_O_4_: La^3+^ nanomaterial.

The antibacterial efficiency of La compounds has been investigated in numerous researches^52,53^. Although a variety of potential antibacterial mechanisms, including the release and penetration of metal ions from the nanoparticle into cells and production of reactive species from the surface of the compound have been proposed, but the actual mechanism is still unclear. The outer membrane wall of the bacteria being damaged by reactive species, such as O_2_, OH^−^ and H_2_O_2_, has been described in numerous research^54^. Recent research demonstrated the antibacterial activity against the bacteria is due to the release of O_2_ radical from the surface of the compound. These free radicals deactivate cellular enzymes and disrupt plasma membrane permeability. When the plasma membrane is disturbed, ROS are released, which can harm proteins and DNA, ultimately cause cell death (Fig. 18). Furthermore, after passing through the cell membrane, metal ions interact with the functional groups of proteins and nucleic acids disrupting enzyme activity and physiological functions that eventually prevent bacterial cell growth^55^.

## Conclusion

The structural investigations of synthesized UCF and LCF NMs from renewable bio-fuel assisted combustion process were well defined by P-XRD, SEM-EDAX, TEM, FT-IR, BET and DRS spectral techniques. The electrochemical studies showed an enhanced capacitance of LCG (26 Ω) than those of UCG (175 Ω) material examined by CV analysis. The synthesized LCG electrode showed enhanced sensor activities for detection of lead heavy metal confirmed by impact of higher reduction potential peak intensities found at + 0.88 & − 0.503 V and the reduction–oxidation-peak potentials were observed at + 0.64 V and − 0.012 V respectively for paracetamol drug chemical. These prepared photocatalysts were shown effective catalytic activity on FOR (20 ppm) dye degradation monitored by UV–Vis spectrophotometry and noted its excellent degradation (91.7%) at 90 min using 40 mg of synthesized samples under Sun-light irradiation. Further, the antibacterial activity of synthesized NMs was investigated and confirms that LCF NMs have higher activity towards gram positive bacteria with an average inhibition zone of 19 mm.

## Data Availability

All data generated or analyzed during this study are included in this published article. We confirm that all the experimental research and field studies on plants (either cultivated or wild), including the collection of plant material, complied with relevant institutional, national, and international guidelines and legislation.

## References

[1] Goldman A (2006). Modern FerriteTechnology.

[2] McBain SC, Yiu HH, Dobson J (2008). Magnetic nanoparticles for gene and drug delivery. Int. J. Nanomed..

[3] Valenzuela R (2012). Novel applications of ferrites. Phys. Res. Int..

[4] Hosseini SA, Niaei A, Salari D, Alvarez-Galvan MC, Fierro JLG (2014). Study of correlation between activity and structural properties of Cu-(Cr, Mn and Co)2 nano mixed oxides in VOC combustion. Ceram. Int..

[5] Ko TH, Lei D, Balasubramaniam S, Seo M-K, Chung Y-S, Kim H-Y, Kim B-S (2017). Polypyrrole-decorated hierarchical NiCo_2_O_4_ nanoneedles/carbon fiber papers for flexible high-performance supercapacitor applications. Electrochim. Acta.

[6] Li J, Xiong D, Wang L, Hirbod MKS, Li X (2019). High-performance self-assembly MnCo_2_O_4_ nanosheets for asymmetric supercapacitors. J. Energy Chem..

[7] Zhu Y, Wang J, Wu Z, Jing M, Hou H, Jia X, Ji X (2015). An electrochemical exploration of hollow NiCo_2_O_4_ submicrospheres and its capacitive performances. J. Power Sources.

[8] Saravanakumar B, Thiyagarajan K, Alluri NR, SoYoon S, Taehyun K, Lin Z-H, Kim S-J (2015). Fabrication of an eco-friendly composite nanogenerator for self-powered photosensor applications. Carbon.

[9] Hu J, Li M, Lv F, Yang M, Tao P, Tang Y, Liu H, Lu Z (2015). Heterogeneous NiCo_2_O_4_@polypyrrole core/sheath nanowire arrays on Ni foam for high performance supercapacitors. J. Power Source.

[10] Surendra BS, Veerabhdraswamy M, Anantharaju KS, Nagaswarupa HP, Prashantha SC (2018). Green and chemical engineered CuFe_2_O_4_: Characterization, cyclic voltammetry, photocatalytic and photoluminescent investigation for multifunctional applications. J. Nanostruct. Chem..

[11] Surendra BS (2018). Green engineered synthesis of Ag-doped CuFe_2_O_4_: Characterization, cyclic voltammetry and photocatalytic studies. J. Sci. Adv. Mater. Dev..

[12] Shetty K, Nagaswarupa HP, Rangappa D, Anantharaju KS, Kumar A (2018). Comparison study of solgel and combustion method for synthesis nano spinel MgFe_2_O_4_ and its influence on electrochemical activity. J. Mater. Today Proc..

[13] Madanakumara H, Jayanna HS, Yelamaggad CV, Soundeswaran S, Vishwas M, Shamala KS, Surendra BS, Basavaraju N (2022). Enhanced electrochemical sensor and photodegradation of industrial wastewater by Almond gum assisted synthesis of Bi_2_O_3_/MgO/Fe_2_O_3_ nanocomposites. Sens. Int..

[14] Gurushantha K, Swetha BN, Chinnam S, Keshavamurthy K, Meena S, Malini S, Roopa KP (2023). Structural, optical, photocatalytic, and antimicrobial attributes of niobium substituted copper nanoferrites. Inorg. Chem. Commun.

[15] Ali KSA, Mohanavel V, Gnanavel C (2021). Structural and optical behavior of SnS_2_/NiFe_2_O_4_ NCs prepared via novel two-step synthesis approach for MB and RhB dye degradation under sun light irradiation. Res. Chem. Intermed..

[16] Dharmaraja C, EmmanuelNicholas P, Ramya P, Isaac Premkumar IJ, Vijayan V, Senthilkumar N (2021). Investigation on photocatalytic activity of ZnS/NiFe_2_O_4_ NCs under sunlight irradiation via a novel two-step synthesis approach. Inorg. Chem. Commun..

[17] Sangeetha M, Senthil TS, Senthilkumar N, Kang M (2022). Solar-light-induced photocatalyst based on Bi–B co-doped TiO_2_ prepared via co-precipitation method. J. Mater. Sci..

[18] Mark JAM, Venkatachalam A, Pramothkumar A, Senthilkumar N, Jothivenkatachalam K, Jesuraj J (2021). Investigation on structural, optical and photocatalytic activity of CoMn_2_O_4_ nanoparticles prepared via simple co-precipitation method. Physica B.

[19] Chennimalai M, Senthil TS, Kang M (2021). A novel green-mediated approach of 3-D hierarchical-like ZnO@Ag, ZnO@Au and ZnO@Ag@Au NCs prepared via *Opuntia ficus indica* fruits extract for enhancement of biological activities. Appl. Phys. A.

[20] GiridharMeenakshi BC, Manjunath SC, Prashantha T, Prashanth BS (2023). Surendra, Super capacitor, electrochemical measurement and sun light driven photocatalytic applications of CuFe_2_O_4_ NPs synthesized from bio-resource extract. Sens. Int..

[21] LakshmiRanganatha V, Pramila S, Nagaraju G, Udayabhanu G, Mallikarjunaswamy C (2020). Cost-effective and green approach for the synthesis of zinc ferrite nanoparticles using Aegle Marmelos extract as a fuel: Catalytic, electrochemical, and microbial applications. J. Mater. Sci..

[22] Raghavendra N, Nagaswarupa HP, Shekhar TRS, Mylarappa M, Prashantha SC, Basavaraju N, RaviKumar CR, AnilKumar MR (2021). Electrochemical sensor studies and optical analysis of developed clay based CoFe_2_O_4_ ferrite NPs. Sens. Int..

[23] Ahmed MA, Okasha N, Oaf M, Kershi RM (2007). The role of Mg substitution on the microstructure and magnetic properties of Ba Co Zn W-type hexagonal ferrites. J. Magn. Magn. Mater..

[24] Almessiere MA, Ünal B, Slimani Y, Korkmaz AD, Baykal A, Ercan I (2019). Electrical properties of La^3+^ and Y^3+^ ions substituted Ni_0.3_Cu_0.3_Zn_0.4_Fe_2_O_4_ nanospinel ferrites. Results Phys..

[25] Dinamani M, Surendra BS, AnandaMurthy HC, Basavaraju N, Shanbhag VV (2023). Green engineered synthesis of Pb_x_Zn_1__−__x_O NPs: An efficient electrochemical sensor and UV light-driven photocatalytic applications. Environ. Nanotech. Monit. Manag..

[26] Haija MA, Basina G, Banat F, Ayesh AI (2019). Adsorption and gas sensing properties of CuFe_2_O_4_ nanoparticles. Mater. Sci..

[27] Rai R, Verma K, Sharma S, Nair SS, Valentec MA, Kholkina AL, Sobolev NA (2011). Structure and magnetic properties of Cd doped copper ferrite. J. Alloys Compds..

[28] Zhu X, Hope-Weeks LJ, Ramirez D, Baghi R, Charles VR, He Y (2019). Controllable decomposition of lanthanum oxychloride through different annealing conditions. J. Alloys. Compds..

[29] Mugutkar AB, Gore SK, Tumberphale UB, Jadhav VV, Mane RS, Patange SM, Shaikh SF, Al-Enizi MUAM, Jadhav SS (2020). The role of La3+ substitution in modification of the magnetic and dielectric properties of the nanocrystalline Co-Zn ferrites. J. Magn. Magn. Mater..

[30] Surendra BS, Veerabhadraswamy M (2017). Microwave assisted synthesis of polymer via bioplatform chemical intermediate derived from Jatropha deoiled seed cake. J. Sci. Adv. Mater. Dev..

[31] Surendra BS, Veerabhadraswamy M (2017). Microwave assisted synthesis of Schiff base via bioplatform chemical intermediate (HMF) derived from Jatropha deoiled seed cake catalyzed by modified Bentonite clay. J Mater. Today Proc..

[32] Salavati-Niasari M, Davar F, Mahmoudi T (2009). A simple route to synthesize nanocrystalline nickel ferrite (NiFe_2_O_4_) in the presence of octanoic acid as a surfactant. Polyhedron.

[33] Aravind G, Raghasudha M, Ravinder D (2015). Electrical transport properties of nano crystalline Li–Ni ferrites. J. Mater..

[34] Gozuak F, Koseoglu Y, Baykal A, Kavas H (2009). Synthesis and characterization of CoxZn_1__−__x_Fe_2_O_4_ magnetic nanoparticles via a PEG-assisted route. J. Magn. Magn. Mater..

[35] Kubelka P, Munk F (1931). Ein beitrag zur optik der farbanstriche. Z. Tech. Phys..

[36] Mahadeva Swamy M, Surendra BS, Mallikarjunaswamy C, Pramila S, Rekha ND (2021). Bio-mediated synthesis of ZnO nanoparticles using Lantana Camara flower extract: Its characterizations, photocatalytic, electrochemical and anti-inflammatory applications. Environ. Nanotech. Monit. Manag..

[37] Surendra BS, Nagaswarupa HP, Hemashree MU, JaveriaKhanum I (2020). Jatropha extract mediated synthesis of ZnFe_2_O_4_ nanopowder: Excellent performance as an electrochemical sensor, UV photocatalyst and an antibacterial activity. J. Chem. Phys. Lett..

[38] Surendra BS, Kiran T, Chethana MV, Savitha HS, Paramesh MS (2021). Cost-effective *Aegle **marmelos* extract-assisted synthesis of ZnFe_2_O_4_:Cu^2+^ NPs: Photocatalytic and electrochemical sensor applications. J Mater. Sci. Mater. Electron..

[39] Raghavendra N, Surendra BS, Shravana Kumar KN, Kantharjau S (2022). Electrochemical, photocatalytic and sensor studies of clay/MgO nanoparticles. Appl. Surf. Sci. Adv..

[40] Basavaraju N, Prashantha SC, Nagabhushana H (2021). Luminescent and thermal properties of novel orange–red emitting MgNb_2_O_6_:Sm^3+^ phosphors for displays, photo catalytic and sensor applications. SN Appl. Sci..

[41] Raghavendra N, Nagaswarupa HP, Shashi Shekhar TR, Mylarappa M, Surendra BS, Prashantha SC, Ravikumar CR, Anil Kumar MR, Basavaraju N (2021). Development of clay ferrite nanocomposite: Electrochemical, sensors and photocatalytic studies. Appl. Surf. Sci. Adv..

[42] Hegde SS, Bose RSC, Surendra BS, Vinoth S, Murahari P, Ramesh K (2022). SnS-Nanocatalyst: Malachite green degradation and electrochemical sensor studies. Mat. Sci. Eng. B.

[43] Basavaraju N, Prashantha SC, Surendra BS, Shashi Shekhar TR, Anil Kumar MR, Ravikumar CR, Raghavendra N, Shashidhara TS (2021). Structural and optical properties of MgNb_2_O_6_ NPs: Its potential application in photocatalytic and pharmaceutical industries as sensor. Environ. Nanotech. Monit. Manag..

[44] Uma B, Anantharaju KS, Renuka L, Nagabhushana H, Malini S, More SS, Vidya YS, Meena S (2020). Controlled synthesis of (CuO-Cu_2_O)Cu/ZnO multi oxide nanocomposites by facile combustion route: A potential photocatalytic, antimicrobial and anticancer activity. Ceram. Int..

[45] Kurlla P, Shivram AK, Kottam N, Siddegowda SB, Subramaniam M, Bogegowda U, Subramanya M, Chowdhury AP, Narasimhan RL (2023). Green-engineered synthesis of Bi2Zr2O7 NPs: Excellent performance on electrochemical sensor and sunlight-driven photocatalytic studies. Environ. Sci. Pollut. Res..

[46] Surendra BS, Gurushantha K, Anantharaju KS, Rudresh M, Basavaraju N, Raghavendra N, Jahagirdar AA, Somashekar HM, Ananda Murthy HC (2023). Effective paracetamol sensor activity, thermal barrier coating (TBC), and UV-light-driven photocatalytic studies of ZrxO_2_: Mg 2+(1–x) nanoparticles. New J. Chem..

[47] Saleh R, Febiana Djaja N (2014). UV light photocatalytic degradation of organic dyes with Fe-doped ZnO nanoparticles. Superl. Microstruct..

[48] Saravanan R, Karthikeyan S, Gupta VK, Sekaran G, Narayanan V, Stephen A (2013). Enhanced photocatalytic activity of ZnO/CuO nanocomposite for the degradation of textile dye on visible light illumination. Mater. Sci. Eng. C..

[49] Fang H, Guo Y, Wu T, Liu Y (2018). Biomimetic synthesis of urchin-like CuO/ZnO nanocomposites with excellent photocatalytic activity. New J. Chem..

[50] Mengting Z, Kurniawan TA, Fei S, Ouyang T, Othman MHD, Rezakazemi M (2019). Applicability of BaTiO_3_/graphene oxide (GO) composite for enhanced photodegradation of methylene blue (MB) in synthetic wastewater under UV–Vis irradiation. Environ. Pollut..

[51] Hegde SS, Surendra BS, Talapatadur V, Murahari P, Ramesh K (2020). Visible light photocatalytic properties of cubic and orthorhombic SnS nanoparticles. Chem. Phys. Lett..

[52] Matsumoto T, Sunad K, Nagai T, Isobe T, Matsushita S, Ishiguro H, Nakajima A (2020). Effects of cerium and tungsten substitution on antiviral and antibacterial properties of lanthanum molybdate. Mater. Sci. Eng. C.

[53] De D, Mandal SM, Gauri SS, Bhattacharya R, Ram S, Roy SK (2010). Antibacterial effect of lanthanum calcium manganate (La0_67Ca0_33MnO3) nanoparticles against *Pseudomonas aeruginosa* ATCC 27853. J. Biomed. Nanotech..

[54] Manikandan A, Manikandan E, Meenatchi B, Vadivel S, Jaganathan SK, Ladchumananandasivam R, Henini M, Maaza M, Aanand JS (2023). Rare earth element (REE) lanthanum doped zinc oxide (La: ZnO) nanomaterials: Synthesis structural optical and antibacterial studies. J. Alloys Comp..

[55] Suwanboon S, Amornpitoksuk P, Bangrak P, Muensit N (2013). Structural, optical and antibacterial properties of nanocrystalline Zn_1-x_La_x_O compound semiconductor. Mater. Sci. Semicond. Process..

